# The recent trend of twin epidemic in the United States: a 10-year longitudinal cohort study of co-prescriptions of opioids and stimulants

**DOI:** 10.1016/j.lana.2025.101030

**Published:** 2025-02-17

**Authors:** Seungyeon Lee, Wenyu Song, David W. Bates, Richard D. Urman, Ping Zhang

**Affiliations:** a Department of Computer Science and Engineering, The Ohio State University, Columbus, OH, USA; b Department of Biomedical Informatics, The Ohio State University, Columbus, OH, USA; c Department of Medicine, Brigham and Women's Hospital, Harvard Medical School, Boston, MA, USA; d Department of Anesthesiology, College of Medicine, The Ohio State University, Columbus, OH, USA

**Keywords:** Twin epidemic, Opioid crisis, Trajectory analysis, Co-prescription pattern, Drug overdose, Patient safety, Stimulant prescription

## Abstract

**Background:**

In recent years, the use of central nervous system stimulant medications has increased among the population already using opioids, referred to as a “twin epidemic.” There is an increasing concern about its harmful outcomes in large populations. However, very few studies examined the co-prescription pattern of these two drug categories over a long period, and there is currently no clear restriction on stimulant prescriptions among patients under opioid treatment in the United States. The objectives of our study were to identify opioid prescription dosage time-dependent patterns and patient subgroups representing distinct trajectories on a national level in the recent 10 years, and to further investigate longitudinal associations between stimulant and opioid prescriptions and the impact of stimulant prescriptions on opioid dosage patterns.

**Methods:**

We obtained patient records from MarketScan, one of the largest clinical databases of health insurance in the United States. 10 years (2012–2021) of prescription records and related patient profiles, who received at least two independent opioid prescriptions, were utilized for developing a group-based opioid dose trajectory model.

**Findings:**

From an initial cohort including 22 million patients with 96 million opioid prescriptions, we developed a study cohort of 2,895,960 patients with a mean age of 43.9 years (standard deviation [SD] 13.0), of whom 1,244,077 (43%) were male. Significant geographical variations in opioid prescription frequency and dosage among four U.S. regions were observed. The trajectory model identified five distinct opioid dose groups. Stimulant prescription before the initial opioid prescription was positively associated with escalating opioid doses (odds ratio [OR]: 7.58; 95% confidence intervals [CI] 6.14–9.35, opioid dose increasing group compared to the decreasing group). Stimulant co-prescriptions were also associated with increasing opioid doses (OR: 1.73; 95% CI 1.40–2.14) and were identified in patients with a higher prevalence of opioid use disorder.

**Interpretation:**

During the recent 10 years, stimulant prescription is positively associated with escalating opioid prescription activities in U.S. healthcare systems, suggesting co-prescriptions of these two types of drugs are an important contributing factor for a national-level twin epidemic. Healthcare leaders and policymakers should pay more attention to this issue and its potential harms.

**Funding:**

10.13039/100000057National Institute of General Medical Sciences, 10.13039/100000026National Institute on Drug Abuse, and 10.13039/100000001National Science Foundation.

**Research in context**

**Evidence before this study**

In recent years, the use of central nervous system stimulant medications has increased among the population with opioid exposure, leading to what is referred to as a “twin epidemic.” The co-use of opioids and stimulants can potentially increase the risk of overdose and other adverse health outcomes, such as cardiovascular events, mental health problems, and even death. We searched PubMed on February 2024 using the terms (“opioid”) and (“dose” or “dosage”) and (“trajectory”) within the past 10 years. The search retrieved 110 studies, but only 15 were relevant to our research. These studies investigated opioid dosage patterns and examined the association between these patterns and the risk of overdose, death, opioid use disorders, and other comorbidities. With the additional term (“stimulant”), only two studies were found, neither of which was pertinent to associations between stimulants and opioids. Furthermore, we found no national-level evidence examining longitudinal associations between opioid and stimulant prescriptions, nor the impact of stimulant prescribing on opioid dosage trajectories. The relevant literature has typically focused solely on opioid doses and is often constrained to short-term studies, single sites, or small populations.

**Added value of this study**

Previous studies have suggested an increasing trend of co-use of these two types of drugs in patient populations, while no large-scale studies have confirmed this pattern on a national level. In this study, we examined longitudinal co-prescription patterns of opioid and stimulant drugs across U.S. healthcare systems and identified a significant positive correlation. Our results, for the first time, provided strong evidence of the emerging twin epidemic in the United States. Healthcare leaders and policymakers should pay more attention to this issue and its potential harms.

**Implications of all the available evidence**

Previous evidence has shown that the co-use of opioids and stimulants could synergistically reinforce dopamine signals and prolong the action of dopamine neuronal activities, leading to euphoric effects. Despite the potential enhanced abuse risk, there is no systematic regulation to guide or restrict co-prescription activities. Our results highlight the severity and prevalence of this issue in healthcare systems and the urgent need for developing corresponding prescription regulations. Our future research will further reveal potential causality mechanisms between co-prescription patterns and patient adverse outcomes.

## Introduction

The opioid epidemic refers to the widespread misuse and abuse of prescription opioids, such as oxycodone, hydrocodone, and fentanyl, as well as illicit opioids, such as heroin.^1^^,^^2^ This epidemic began in the late 1990s and has since resulted in a dramatic increase in opioid-related overdose deaths. In 2023, there were 81,083 opioid overdose deaths in the United States.^3^

In recent years, there has been a significant increase in both opioid- and central nervous system stimulant medication-related overdose deaths.^4^ The opioid and stimulant epidemics are two intertwined public health crises that have been described as the “twin epidemic.”^5^, ^6^, ^7^, ^8^ Both epidemics involve the misuse and abuse of prescription and illicit drugs, and both have led to a significant increase in overdose deaths and other adverse health outcomes.^9^^,^^10^ The twin epidemic has emerged due to the overlap between the two epidemics. Many individuals who misuse opioids also use stimulants, and vice versa.^11^ The co-use of opioids and stimulants can increase the risk of overdose and other adverse health outcomes, such as cardiovascular events, mental health problems, and even death.^12^, ^13^, ^14^ The twin epidemic of opioid and stimulant abuse is particularly concerning because these drugs exert different effects on the body and can interact in unpredictable ways.^15^ Addressing the twin epidemic requires a comprehensive approach that includes prevention, treatment, and harm reduction strategies.^16^ These strategies should be tailored to the specific needs of each individual who is affected by the twin epidemic.

Despite the wealth of evidence supporting the potential enhanced abuse risk of co-prescription of stimulant and opioid drugs and the need for tailored strategies for individuals who might experience the twin epidemic, there have been no large-scale studies to evaluate longitudinal associations between stimulant prescription and opioid prescription trajectories, across U.S. healthcare systems. One study reported increased risk of overdose with co-use of opioids and stimulants^12^ was solely based on provincial health records from Canada. The lack of evidence creates challenges for policymakers to initiate corresponding regulations to address potential harms with a systematic manner. Previous work has mainly focused on investigating opioid dosage trajectories only,^17^, ^18^, ^19^ and was often limited to short-term, single site or small populations.

The objectives of our study were to identify opioid prescription dosage time-dependent patterns and patient subgroups representing distinct trajectories on a national level in the recent 10 years, and to further investigate longitudinal associations between stimulant and opioid prescriptions and the impact of stimulant prescriptions on opioid dosage patterns. Additionally, we investigated regional differences in opioid and stimulant prescribing patterns among four major regions of the U.S. to provide insights essential for developing targeted, region-specific policies to more effectively address the opioid and twin epidemics.

## Methods

### Study design

In this retrospective cohort study, we used large-scale longitudinal patient-level healthcare data from the MarketScan Commercial Claims and Encounters (CCAE), which provides some of the largest convenience samples available in proprietary U.S. databases—with over 293 million unique patients since 1995. The database contains de-identified, individual-level healthcare claims on a national scale and is primarily used to evaluate health utilization and services.

We identified approximately 20 million patients with around 96 million opioid prescription records in the MarketScan database from Jan 1, 2012, to Dec 31, 2021. Of these, about 2.8 million patients met our study cohort selection criteria. Each patient had a comprehensive set of medical records, encompassing demographic characteristics, drug prescriptions, diagnoses, procedures, and other clinically relevant indicators with time information. The data utilized for this study included patient demographics, diagnosis, and drug prescription claims. Diagnoses and drug codes are defined according to the International Classification of Diseases (ICD-9/10) and National Drug Codes (NDC), respectively. Drug records include the number and type of drugs dispensed, the route of administration, days' supply, and the strength per unit. We included only non-missing records in these fields. No substantial missingness was observed across all regions and age groups (126,099/97,049,173 [0.1%]), suggesting completeness of the dataset and a very small amount of missing at random.

### Participants

The study cohort included all individuals who received at least two independent opioid prescriptions at different times. Patient eligibility required at least 12 months of continuous enrollment before the index date—the date of the first opioid prescription—to establish the baseline period, and at least two months of continuous enrollment after the index date to define the follow-up period. Individuals were excluded if they were younger than 18 years on the index date or had been diagnosed with any cancer. Additionally, those who had been prescribed buprenorphine before the eligible opioid prescriptions were excluded, as buprenorphine is primarily used to treat opioid use disorder.^19^^,^^20^ The study period begins at the index date and follow-ups for each individual ended after 9 years or upon loss to follow-up (the end of the continuous enrollment), whichever occurred first. The number of included and excluded patients is detailed in Supplementary Figure S1.

Opioids included in the study were codeine, dihydrocodeine, hydrocodone, hydromorphone, levorphanol, meperidine, methadone, morphine, nalbuphine, opium, oxycodone, oxymorphone, pentazocine, propoxyphene, remifentanil, and sufentanil (Supplementary Table S1). We included only oral prescriptions, and excluded patches, injections, and other routes. Opioid and stimulant drugs were identified using the National Drug Codes, including their combination with other drug classes. The list of stimulants included in the study is provided in Supplementary Table S1.

### Study variables

All opioid prescriptions were converted to oral morphine milligram equivalents (MMEs). We determined the patient's daily dose of opioids in MME (mg/day) by multiplying the strength and quantity of opioids dispensed on the day by published conversion factors, expressed as: strength per unit × (number of units/days supplied) × MME conversion factor. We calculated the monthly MME of all opioids dispensed for every non-overlapped 30-day period from the index date at the individual patient level and divided each by 30 days to obtain the mean daily MME.

We modeled the MMEs over time at monthly intervals. The primary covariate was time, defined as the relative time span over which opioid prescriptions were monitored. We incorporated stimulant prescriptions as a time-varying covariate to control for potential confounding factors that could influence the trajectories. At each time step, we calculated the cumulative number of stimulant prescriptions, spanning from one year before the index date up to that time step, and treated it as a time-varying covariate.

### Cohort characteristics

We explored cohort characteristics to investigate their associations with opioid dose trajectory groups. Demographic characteristics included age on the index date, sex, and region. We used patients' regional information to compare opioid prescribing practices by region, grouped into four major regions: Northeast, North Central, South, and West.

Characteristics related to opioid dispensing included the number of opioid prescriptions, the Avg MME, and the Total MME. The number of opioid prescriptions was defined as the number of months in which opioids were dispensed over the study period, with mean and median values calculated across all patients. The Avg MME was defined as the average of the mean daily MMEs for months in which opioids were dispensed, and the Total MME was computed as the sum of all the mean daily MMEs. For a stimulant characteristic, we assessed the total number of stimulant prescriptions each patient received from their first to last opioid prescription dates.

Comorbidities were also assessed as the cohort characteristics, as several studies have suggested a recent increase in stimulant prescriptions that overlap with opioid prescriptions, which is often related to treating attention deficit hyperactivity disorder (ADHD), depression, and other comorbidities in opioid use disorder (OUD) patients or chronic pain in attention deficit hyperactivity disorder patients.^10^ The comorbidities included attention deficit hyperactivity disorder, mental health disorders, such as depression, anxiety, schizophrenia/psychoses, and bipolar, and substance use disorders, involving opioid, tobacco, alcohol, cannabis, and other substances. All comorbidities were identified using ICD-9/10 codes (Supplementary Table S2) at two different times: after the index date and within one year prior to the index date.

### Opioid trajectory modeling

We employed grouped-based trajectory modeling (GBTM)^21^ to identify multiple distinct longitudinal trajectories of opioid prescription dosage over time and classify the cohort into trajectory groups. We analyzed the trajectories of monthly opioid doses over a 9-year period after cohort entry. A variety of model configurations were explored, with the best model selected using the Bayesian information criterion (Supplementary Table S12).

### Statistical analysis of covariates

Patient groups by region, gender, stimulant prescriptions, and trajectory groups were compared statistically using Kruskal–Wallis and Mann–Whitney U tests, with significance defined as *P* < 0.05. The characteristics compared included age, the number of opioid prescriptions, the Avg MME, and the Total MME.

We employed a multivariate logistic regression model estimating trajectory membership to compute odds ratios (OR) between different trajectory groups,^19^ evaluating factors associated with each group based on patient demographics, comorbidities within one year prior to the index date, and stimulant prescription characteristics.

Detailed descriptions of the methods, including study variables, trajectory modeling, and statistical analyses, are provided in the Supplementary Methods. Statistical analyses were performed using the SciPy package in Python (version 3.9.7). We followed STROBE reporting guidelines (Supplementary Table S13).

### Ethics committee approval

This study used secondary, de-identified data and was therefore exempt from ethics committee approval.

### Role of the funding source

Funders had no role in the study design, data collection, data analysis, data interpretation, or writing of the manuscript.

## Results

### Descriptive summary of study cohort and geographical variations of opioid prescriptions

From an initial cohort of 22,586,656 patients, we developed a study cohort with 2,895,960 patients (Table 1 and Supplementary Figures S2 and S3). The cohort selection pipeline is summarized in Supplementary Figure S1. The average length of follow-up for all patients was 1277 days (standard deviation [SD]: 879). The median age at the index date was 46 years (interquartile range [IQR] 34–55). The median number of opioid prescriptions was 3, and the Avg MME had a median of 5.42. We observed a higher proportion of comorbidities following opioid exposure compared to before exposure. Notably, the percentage for opioid use disorder after the index date was 5 times higher (0.2% vs 1.1%), and the percentages for mental health disorders as well as other substance use disorders were more than twice as high.

**Table 1:** Summary statistics of the study cohort.

	Measure	Value
Demographics	Patient No	2,895,960
	Female (%)	1,651,883 (57.0)
	Male (%)	1,244,077 (43.0)
	Age, median	46 (34–55)
Opioid Prsc	Prsc No, mean	4.01 (5.82)
	Prsc No, median	3 (2–4)
	Avg MME, mean	9.80 (16.73)
	Avg MME, median	5.416 (3.58–9.17)
	Total MME, mean	57.13 (278.89)
	Total MME, median	15 (8.83–31.66)
Stimulant Prsc	Patient^a^ No (%)	160,243 (5.5)
	Prsc No^b^, mean	12.44 (16.28)
	Prsc No, median	6 (2–16)
Comorbidities^c^	Depression (%)	281,463 (9.7)
	Anxiety	352,879 (12.2)
	Bipolar	56,612 (1.9)
	Schizophrenia/Psychoses	7668 (0.3)
	Chronic Acute Pain	1,669,571 (57.7)
	ADHD	76,217 (2.6)
	Opioid overdose	785 (0.03)
	Opioid use disorder	6397 (0.2)
	Tobacco use disorder	180,710 (6.2)
	Alcohol use disorder	33,638 (1.2)
	Cannabis use disorder	9939 (0.3)
	Other substance use disorder	12,983 (0.4)
Comorbidities^d^	Depression (%)	591,356 (20.4)
	Anxiety	881,093 (30.4)
	Bipolar	142,969 (4.9)
	Schizophrenia/Psychoses	18,992 (0.7)
	Chronic Acute Pain	2,325,814 (80.3)
	ADHD	136,857 (4.7)
	Opioid overdose	5280 (0.2)
	Opioid use disorder	31,597 (1.1)
	Tobacco use disorder	372,575 (12.9)
	Alcohol use disorder	90,460 (3.1)
	Cannabis use disorder	33,543 (1.2)
	Other substance use disorder	42,070 (1.5)

Data are n (%), median (IQR), or mean (SD). No = number, Prsc = prescription, IQR = interquartile range, Avg MME = the average of the mean daily MMEs for months in which opioids were dispensed, Total MME = the sum of all the mean daily MMEs, ADHD = Attention-deficit/hyperactivity disorder.

a Patients with stimulant prescriptions were identified based on whether they were prescribed stimulants at any time between the start and end dates of their opioid prescriptions.

b Stimulant Prsc No was measured between the start and end dates of opioid prescriptions for each individual patient.

c Comorbidities were assessed within one year prior to the index date.

d Comorbidities were assessed after the index date.

Significant variation in opioid prescription dosage and numbers was identified among four U.S. regions: South, North Central, West, and Northeast (Table 2 and Supplementary Table S3). Both the South and the West groups had higher Total MME values of 59.31 and 62.66, respectively, compared to the overall study cohort, as well as the Northeast and North Central. The South showed the lowest Avg MME of 8.99 but the highest number of opioid prescriptions of 4.19. In contrast, the West had the highest Avg MME of 12.93, but its number of opioid prescriptions at 3.80 was below the overall average. The South included the highest percentage of patients with stimulant prescriptions at 6.2% (Supplementary Table S3). The results of statistical tests comparing regions are presented in Supplementary Table S11.

**Table 2:** Comparison of patient subgroups by region, gender, and stimulant prescriptions.

	Region				Gender			Stimulant Prsc^a^		
	Northeast	North Central	South	West	Male	Female	*P* value^c^	w/o STM	w/STM	*P* value^c^
**Demographics**										
Patient No (%)	370,408 (12.8)	584,685 (20.2)	1,440,593 (49.7)	469,963 (16.2)	1,244,077 (43.0)	1,651,883 (57.0)	–	2,735,717 (94.5)	160,243 (5.5)	–
Age, mean	44.63 (13.04)	44.01 (13.28)	43.79 (12.84)	43.55 (13.09)	45.08 (12.89)	43.04 (13.02)	<0.0001	44.22 (12.95)	38.76 (12.82)	<0.0001
**Opioid Prsc**										
Prsc No (%)	3.70 (5.06)	3.95 (5.47)	4.19 (6.38)	3.80 (5.03)	4.08 (6.08)	3.96 (5.63)	<0.0001	3.92 (5.58)	5.55 (8.86)	<0.0001
Avg MME, mean	9.23 (15.97)	9.52 (15.42)	8.99 (15.03)	12.93 (22.31)	10.60 (17.91)	9.19 (15.75)	<0.0001	9.73 (16.61)	10.90 (18.56)	<0.0001
Total MME, mean	50.49 (255.25)	50.18 (216.05)	59.31 (312.02)	62.66 (256.99)	64.11 (307.66)	51.87 (254.96)	<0.0001	54.51 (264.47)	101.78 (457.67)	<0.0001
**Comorbidities**^b^										
Depression (%)	72,684 (19.6)	130,081 (22.2)	290,205 (20.1)	93,154 (19.8)	168,248 (13.5)	423,108 (25.6)	–	521,355 (19.1)	70,001 (43.7)	–
Anxiety	117,600 (31.7)	181,026 (31.0)	445,309 (30.9)	131,572 (28.0)	277,903 (22.3)	603,190 (36.5)	–	791,531 (28.9)	89,562 (55.9)	–
Bipolar	21,112 (5.7)	31,145 (5.3)	67,851 (4.7)	21,875 (4.7)	45,610 (3.7)	97,359 (5.9)	–	118,157 (4.3)	24,812 (15.5)	–
Schizophrenia	2854 (0.8)	4086 (0.7)	8783 (0.6)	3088 (0.7)	8389 (0.7)	10,603 (0.6)	–	16,387 (0.6)	2605 (1.6)	–
Chronic Pain	304,849 (82.3)	476,720 (81.5)	1,152,298 (80.0)	369,144 (78.5)	991,176 (79.7)	1,334,638 (80.8)	–	2,188,539 (80.0)	137,275 (85.7)	–
ADHD	14,867 (4.0)	26,438 (4.5)	76,764 (5.3)	17,640 (3.8)	54,732 (4.4)	82,125 (5.0)	–	42,838 (1.6)	94,019 (58.7)	–
Opioid overdose	641 (0.2)	1204 (0.2)	2573 (0.2)	831 (0.2)	2214 (0.2)	3066 (0.2)	–	4679 (0.2)	601 (0.4)	–
OUD	4621 (1.2)	5092 (0.9)	17,333 (1.2)	4301 (0.9)	15,717 (1.3)	15,880 (1.0)	–	27,306 (1.0)	4291 (2.7)	–
TUD	47,951 (12.9)	92,296 (15.8)	187,157 (13.0)	41,374 (8.8)	189,768 (15.3)	182,807 (11.1)	–	347,642 (12.7)	24,933 (15.6)	–
AUD	14,689 (4.0)	20,840 (3.6)	38,883 (2.7)	15,480 (3.3)	54,955 (4.4)	35,505 (2.1)	–	81,880 (3.0)	8580 (5.4)	–
CUD	5007 (1.4)	9198 (1.6)	13,343 (0.9)	5841 (1.2)	19,095 (1.5)	14,448 (0.9)	–	29,305 (1.1)	4238 (2.6)	–
Other SUD	6541 (1.8)	7841 (1.3)	21,079 (1.5)	6248 (1.3)	21,565 (1.7)	20,505 (1.2)	–	35,525 (1.3)	6545 (4.1)	–

Data are n (%) or mean (SD). No = number, Prsc = prescription, w/o STM = patients without stimulant prescriptions, w/STM = patients with stimulant prescriptions, Avg MME = the average of the mean daily MMEs for months in which opioids were dispensed, Total MME = the sum of all the mean daily MMEs, ADHD = Attention-deficit/hyperactivity disorder, OUD = opioid use disorder, TUD = tobacco use disorder, AUD = alcohol use disorder, CUD = cannabis use disorder, SUD = substance use disorder.

a Patients with stimulant prescriptions were identified based on whether they were prescribed stimulants at any time between the start and end dates of their opioid prescriptions.

b Comorbidities were assessed after the index date.

c Mann-Whitney U tests were performed to compare patient subgroups by gender and stimulant prescriptions.

When we compared gender groups (Table 2 and Supplementary Table S10), males were older than females, with mean ages of 45.1 and 43.0, respectively, and had higher opioid doses, with the Avg MME values of 10.60 and 9.19, and the Total MME values of 64.11 and 51.87, respectively.

### Model 1: opioid dose trajectory baseline model

**Fig. 1:**
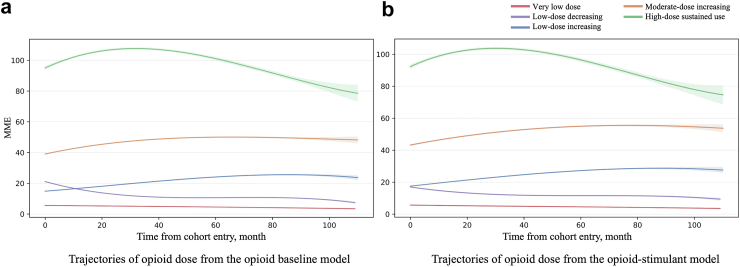
Trajectories of opioid doses from (a) the opioid baseline model and (b) the opioid-stimulant model. ∗The figure shows trajectories of the mean daily MME over 9 years. The shaded areas show 99% confidence intervals.

We identified five distinct trajectories of opioid dosage during 108 months from the opioid baseline model without the stimulant covariate (Fig. 1(a) and Supplementary Table S4): very low-dose (2,243,167 patients, 77.5%) with a median of 4.5 Avg MME (IQR 3.3–6.3), low-dose decreasing (204,607, 7.0%) with 14.3 (IQR 11.2–21.7), low-dose increasing (335,266, 11.6%) with 14.6 (IQR 11.9–18.8), moderate-dose increasing (68,504, 2.4%) with 43.8 (IQR 36.1–55.4), and high-dose sustained use (44,416, 1.5%) with 104.1 (IQR 84.2–137.5).

The very low-dose and high-dose sustained use groups exhibited the largest differences in opioid-related characteristics. The differences were 3-fold for the mean number of opioid prescriptions, with 3.21 in the very low-dose group and 9.66 in the high-dose sustained use group, 23-fold for the Avg MME, with 4.91 and 115.43, and about 54-fold for the Total MME, with 16.40 and 884.60, respectively (Supplementary Table S4).

### Model 2: the associations between stimulant prescriptions and opioid dose trajectories

We obtained patients with stimulant prescriptions who were prescribed stimulants at any point between their first and last opioid prescription dates, totaling 160,243 (5.5%) patients with a median of 6 stimulant prescriptions (IQR 2–16). Patients with stimulant prescriptions were younger than those without, with mean ages of 38.8 and 44.2 years, respectively, and had more opioid prescriptions and higher opioid doses, with mean numbers of opioid prescriptions of 5.55 and 3.92, and the mean Avg MME values of 10.90 and 9.73 (Table 2). The mean Total MME was twice as high in the group with stimulant prescriptions, at 101.78 compared to 54.51. Additionally, the stimulant group had a heavier burden of comorbidities after the index date than those without stimulant prescriptions.

Stimulant prescriptions were included in the opioid trajectory model as a time-varying covariate to evaluate the relationship between stimulant prescriptions and opioid dose trajectories (Fig. 1(b) and Table 3). Trajectory groups were named based on observed dosage trends in Fig. 1(b) as ‘very low,’ ‘low-dose increasing,’ ‘low-dose decreasing,’ ‘moderate-dose increasing,’ and ‘high-dose sustained.’ We observed shifted patient group memberships for the opioid-stimulant model compared to the opioid baseline model.

**Table 3:** Descriptive characteristics by trajectory groups from the opioid-stimulant model.

	Measure	Very low-dose	Low-dose	Low-dose	Moderate-dose	High-dose	*P* value^e^
			Decreasing	Increasing	Increasing	Sustained use	
**Demographics**	Patient No (%)	2,245,684 (77.5)	295,527 (10.2)	245,072 (8.5)	68,248 (2.4)	41,429 (1.4)	–
	Female (%)	1,322,171 (58.9)	151,186 (51.2)	122,151 (49.8)	35,311 (51.7)	21,064 (50.8)	–
	Age, median	44 (33–54)	50 (39–57)	50 (39–58)	48 (37–57)	49 (38–56)	<0.0001
**Opioid Prsc**	Prsc No, mean	3.15 (2.55)	6.34 (8.04)	6.40 (8.28)	10.30 (16.39)	9.92 (15.11)	<0.0001
	Prsc No, median	2 (2–3)	4 (3–7)	3 (2–5)	3 (2–10)	4 (2–9)	–
	Avg MME, mean	4.93 (2.26)	15.33 (6.67)	18.04 (8.43)	51.68 (14.12)	116.08 (42.79)	<0.0001
	Avg MME, median	4.55 (3.25–6.25)	12.77 (10.66–17.5)	16.5 (13.33–21.52)	48.55 (40–60.83)	108.3 (83.33–141.6)	–
	Total MME, mean	15.96 (20.42)	91.16 (136.83)	129.71 (186.81)	489.84 (741.76)	904.02 (1494.84)	<0.0001
	Total MME, median	12 (7.67–18.66)	56.66 (35.26–99.16)	50 (32.5–90.5)	168.3 (107.5–435)	335.8 (228.7–708.3)	–
**Stimulant Prsc**	Patient^a^ No (%)	111,087 (4.9)	14,793 (5.0)	23,616 (9.6)	6681 (9.8)	4066 (9.8)	–
	Prsc No^b^, mean	12.33 (16.94)	15.70 (18.50)	10.88 (16.22)	12.19 (15.89)	13.01 (17.97)	<0.0001
	Prsc No, median	6 (2–16)	8 (3–21)	5 (2–14)	6 (2–15)	7 (2–16)	–
**Comorbidities**^c^	Depression (%)	202,919 (9.0)	32,545 (11.0)	29,636 (12.1)	10,040 (14.7)	6323 (15.3)	–
	Anxiety	265,442 (11.8)	36,079 (12.2)	33,912 (13.8)	10,993 (16.1)	6453 (15.6)	–
	Bipolar	41,772 (1.9)	6010 (2.0)	5744 (2.3)	1924 (2.8)	1162 (2.8)	–
	Schizophrenia/Psychoses	5363 (0.2)	885 (0.3)	910 (0.4)	287 (0.4)	223 (0.5)	–
	Chronic Acute Pain	1,187,764 (52.9)	216,649 (73.3)	186,744 (76.2)	48,310 (70.8)	30,104 (72.7)	–
	ADHD	56,259 (2.5)	5908 (2.0)	10,181 (4.2)	2611 (3.8)	1258 (3.0)	–
	Opioid overdose	501 (0.02)	100 (0.03)	99 (0.04)	52 (0.08)	33 (0.08)	–
	Opioid use disorder	3080 (0.1)	758 (0.3)	1194 (0.5)	833 (1.2)	532 (1.3)	–
	Tobacco use disorder	129,032 (5.8)	21,780 (7.4)	19,272 (7.9)	6555 (9.6)	4071 (9.8)	–
	Alcohol use disorder	22,534 (1.0)	4583 (1.6)	4409 (1.8)	1325 (1.9)	787 (1.9)	–
	Cannabis use disorder	7533 (0.3)	949 (0.3)	920 (0.4)	338 (0.5)	199 (0.5)	–
	Other substance use disorder	8350 (0.4)	1569 (0.5)	1636 (0.7)	831 (1.2)	597 (1.4)	–
**Comorbidities**^d^	Depression (%)	433,467 (19.3)	75,912 (25.7)	52,576 (21.5)	17,744 (26.0)	11,657 (28.1)	–
	Anxiety	670,977 (29.9)	104,314 (35.3)	69,254 (28.3)	22,316 (32.7)	14,232 (34.4)	–
	Bipolar	106,133 (4.7)	18,075 (6.1)	12,133 (5.0)	3961 (5.8)	2667 (6.4)	–
	Schizophrenia/Psychoses	13,425 (0.6)	2576 (0.9)	1861 (0.8)	629 (0.9)	501 (1.2)	–
	Chronic Acute Pain	1,734,406 (77.2)	276,009 (93.4)	219,610 (89.6)	58,738 (86.1)	37,051 (89.4)	–
	ADHD	101,355 (4.5)	12,337 (4.2)	16,583 (6.8)	4250 (6.2)	2332 (5.6)	–
	Opioid overdose	2937 (0.1)	1024 (0.3)	674 (0.3)	361 (0.5)	284 (0.7)	–
	Opioid use disorder	12,956 (0.6)	6113 (2.1)	6189 (2.5)	3869 (5.7)	2470 (6.0)	–
	Tobacco use disorder	270,142 (12.0)	49,778 (16.8)	33,582 (13.7)	11,541 (16.9)	7532 (18.2)	–
	Alcohol use disorder	63,613 (2.8)	13,792 (4.7)	8619 (3.5)	2664 (3.9)	1772 (4.3)	–
	Cannabis use disorder	25,375 (1.1)	4068 (1.4)	2566 (1.1)	906 (1.3)	628 (1.5)	–
	Other SUD	26,706 (1.2)	6493 (2.2)	4791 (2.0)	2355 (3.5)	1725 (4.2)	–

Data are n (%), median (IQR), or mean (SD). Demo = demographics, No = number, Prsc = prescription, IQR = interquartile range, STM = stimulant, Avg MME = the average of all the mean daily MMEs for months in which opioids were dispensed, Total MME = the sum of all the mean daily MMEs, ADHD = Attention-deficit/hyperactivity disorder, SUD = substance use disorder.

a Patients with stimulant prescriptions were identified based on whether they were prescribed stimulants at any time between the start and end dates of their opioid prescriptions.

b Stimulant Prsc No was measured between the start and end dates of opioid prescriptions for each individual patient.

c Comorbidities were assessed within one year prior to the index date.

d Comorbidities were assessed after the index date.

e Kruskal-Wallis tests were performed to compare all trajectory groups.

**Fig. 2:**
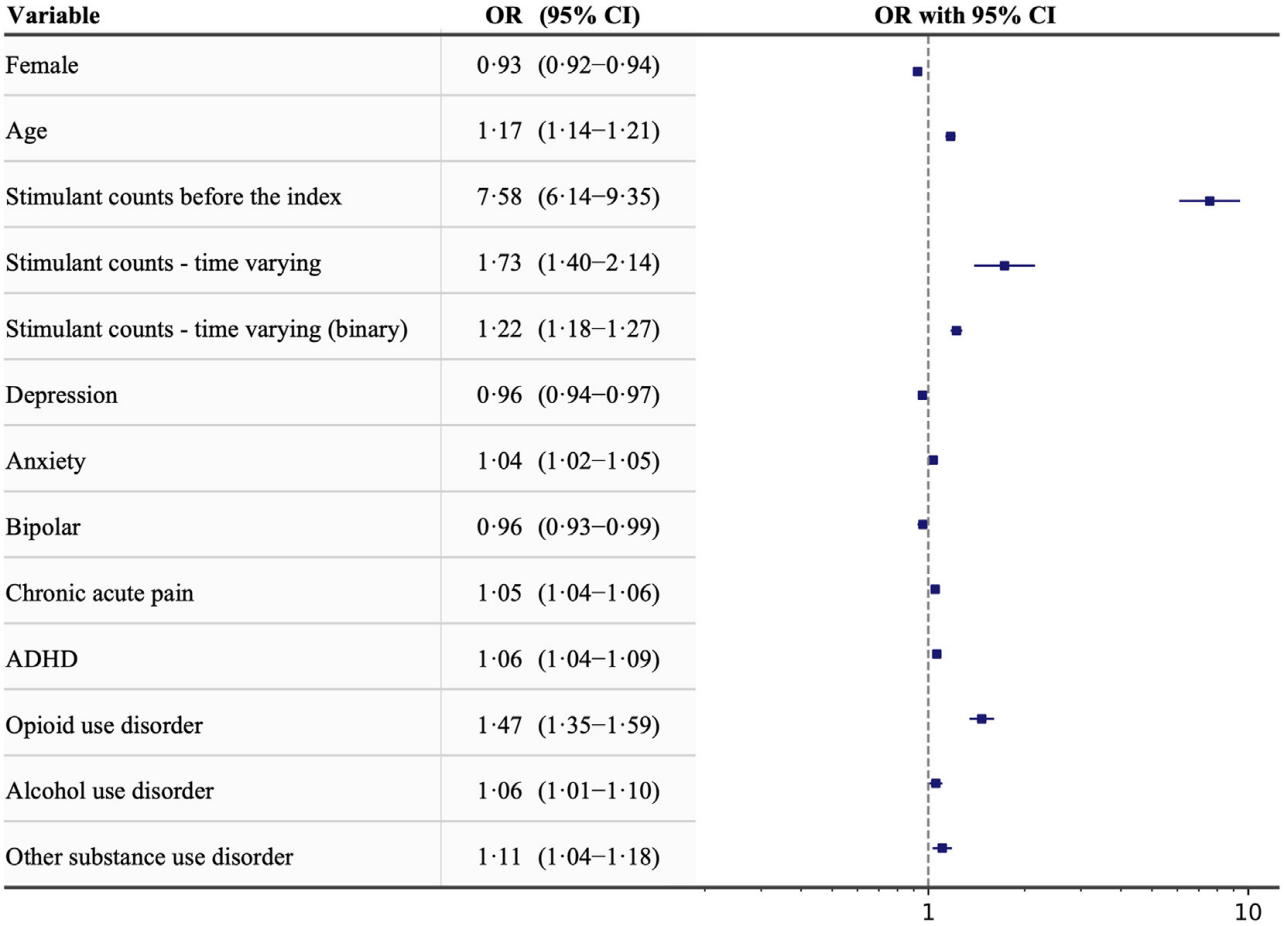
Distribution plots of odds ratio from a logistic regression model estimating trajectory membership for the low-dose decreasing group (reference) vs the low-dose increasing group to evaluate factors associated with the groups. ∗All reported variables were statistically significant with *P* < 0.01. The comorbidities were assessed within one year prior to the index date. Simulant counts before the index date were measured as the total number of stimulant prescriptions within one year prior to the index date. Simulant counts—time varying represented the cumulative number of stimulant prescriptions at each monthly interval time step from one year before the index date up to that time step, while Simulant counts—time varying (binary) represented these counts as binary values (1 if the count is greater than 0, otherwise 0). OR = odds ratio, CI = confidence interval, ADHD = Attention-deficit/hyperactivity disorder.

Among the trajectory groups, patients prescribed stimulants had the highest rates (9.8%) in the moderate-dose increasing and high-dose sustained use groups (Table 3), followed by the low-dose increasing group (9.6%), which were about twice as high compared to the very low-dose (4.9%) and low-dose decreasing groups (5.0%). These three groups also had higher proportions of patients with attention deficit hyperactivity disorder or substance use disorders. In the group comparative analysis (Fig. 2), we focused on the low-dose decreasing and low-dose increasing groups, which initiated opioids at similar doses, to investigate factors associated with the increasing opioid dose trajectory, specifically the impact of stimulant prescriptions on subsequent trajectory patterns. The number of stimulant prescriptions before the index date had a relatively higher odds ratio (OR: 7.58; 95% confidence intervals [CI]: 6.14–9.35) for being in the increasing group compared to the decreasing group. The time-varying number of simulant prescriptions was also associated with a higher odds ratio (OR: 1.73; 95% CI: 1.40–2.14).

Regional variations were also observed among the identified groups. The moderate-dose increasing and high-dose sustained use groups had higher proportions of patients from the West and fewer from the South compared to the overall study cohort and other groups (Supplementary Tables S5–S9).

## Discussion

The so-called twin epidemic has drawn increasing attention in recent years. Despite strict regulations on opioid and stimulant prescriptions, both opioid- and stimulant-related overdose deaths have kept increasing,^4^ and the group with co-prescription is at high risk.^12^, ^13^, ^14^ Studies have suggested that the co-use of opioids and stimulants can synergistically reinforce drug effects and increase the risk of overdose and other adverse health outcomes.^9^^,^^10^ In this study, we conducted one of the largest analyses to examine the potential associations between opioid and stimulant prescriptions over the recent 10 years across the United States. We utilized MarketScan, one of the largest national claims databases, for cohort development and pattern identification, which has been used in multiple studies to investigate clinical patterns across the country.^22^^,^^23^ One of the major aims of this study is to provide data-driven evidence on national prescription trends to facilitate future opioid and stimulant prescription guidelines and interventions. Currently, the United States does not restrict central nervous system stimulant prescriptions among patients with substance use disorder or under opioid treatment, while regulation policy has been implemented in some north European countries. For example, Norway has restrictions on prescribing central stimulants to individuals who have substance use disorders or who are on opioid maintenance treatment.^24^ Similar restrictions could be recommended in US healthcare systems. However, more large-scale studies in US patient populations need to be conducted to support adaption of related prescription guidelines.

Our trajectory model identified five distinct opioid dosage trajectories from the model with the stimulant covariate (Fig. 1(b) and Table 3). Adding stimulant co-prescriptions as a time-varying covariate did change the shape of opioid trajectories and patient group memberships, suggesting a correlation between opioid doses and stimulant prescriptions over time. Specifically, with the time-varying stimulant covariate, the proportion of patients with stimulant prescriptions increased in groups showing increasing trends or moderate-to-high opioid doses (Table 3) compared to the opioid baseline model (Supplementary Table S4). The proportions in the opioid baseline model vs. the opioid-stimulant model are: very low dose (5.3% vs 4.9%), low-dose decreasing (7.6% vs 5.0%), low-dose increasing (5.1% vs 9.6%), moderate-dose increasing (6.7% vs 9.8%), and high-dose sustained use (7.6% vs 9.8%). In Fig. 1, the starting points of the low-dose decreasing and low-dose increasing groups differed between the opioid baseline and opioid-stimulant models, suggesting changes in group membership, which align with the observed variations in the proportions of patients with stimulant prescriptions in these groups.

In the opioid-stimulant model (Table 3), the vast majority of the cohort (77.5%) exhibited very low opioid doses, which is consistent with previous studies.^17^^,^^19^ The very low-dose group exhibited the lowest percentages of patients with most comorbidities before the index date, while the high-dose sustain use group had the highest percentages. The comorbidities present before initiating opioids appear to be associated with higher opioid doses, indicating that underlying health conditions were potentially leading to subsequent opioid prescribing. With this knowledge, screening for comorbidities at the time of first opioid prescribing and at subsequent visits can be essential in managing opioid prescriptions to prevent escalation to high-dose, and even persistent, opioid use.

Patients who were prescribed stimulants had the highest rates in the moderate-dose increasing and high-dose sustained use groups, followed by the low-dose increasing group. Stimulant prescription appears to be associated with higher or increasing opioid dose groups, indicating relations between these two types of drugs in opioid prescription activities. The finding was further supported by statistical analysis of the study cohort (Table 2), which showed that patients prescribed stimulants were more frequently prescribed opioids and had higher doses compared to those without stimulant prescriptions. Stimulant use alongside opioids may contribute to higher or elevated dosage patterns, underscoring the need for careful management of these drugs.

We further quantified associations between stimulant and opioid prescriptions. The number of stimulant prescriptions prior to the initial opioid prescription had a relatively higher odds ratio (OR: 7.58, 95% CI: 6.14–9.35) for being in the low-dose increasing group compared to the decreasing group, which strongly indicates that stimulant prescriptions before initiating opioids are positively associated with escalating opioid doses. The time-varying number of stimulant prescriptions also had a higher odds ratio (OR: 1.73, 95% CI: 1.40–2.14), suggesting that stimulant co-prescriptions were significantly associated with increasing opioid doses, and this could potentially lead to a higher rate of opioid use disorder. This positive association we identified suggested a persistent driving force to the twin epidemic on a national level.

Our study also revealed gender variations in opioid prescribing practices. Males were prescribed opioids more frequently and at higher doses compared to females. The finding is supported by previous evidence showing that males received higher doses of pain medication among patients with chronic pain,^25^ and were more likely to escalate to high-dose opioid therapy than females.^26^ Furthermore, healthcare providers issued fewer prescriptions to females during ambulatory care and outpatient visits.^25^ The difference in opioid prescribing practices likely reflects underlying gender disparities in the management of medical conditions.

We also observed prescription variations among four U.S. regions. The South and the West regions had higher Total MMEs compared to the overall rate of the country, as well as the Northeast and North Central regions. The West had fewer opioid prescriptions but tended to prescribe more potent opioids per prescription. The finding aligns with the previous research indicating that high-dose initial prescriptions were most common among patients in the West, and long-duration prescriptions were most prevalent in the South.^27^ Another study found that healthcare providers in the Northeast and Midwest were significantly less likely to prescribe opioids than those in the South and West.^28^ Importantly, our study duration was based on recent data, suggesting these regional variations persist in healthcare systems. The regional variations may be influenced by the local social environment, the prescribing practices of providers in the region,^27^^,^^28^ and the regulation of healthcare systems. Understanding these regional variations and which approaches achieve better outcomes is crucial for developing future region-specific policies to address the opioid- and twin epidemic more effectively.

This study has several limitations. First, the dataset is de-identified and lacks race information, preventing an analysis of racial differences in opioid prescribing practices. Given the extensive research on racial/ethnic differences in opioid prescribing patterns,^29^ especially in pain management within the U.S. health system,^30^ understanding racial disparities is crucial for addressing inequities in healthcare. Second, the absence of provider and hospital information in our dataset limits our ability to further assess the influence of provider and hospital settings^27^^,^^28^ on prescription patterns. Third, co-prescription of these two categories may be clinically indicated in certain patients, so across-the-board bans of co-prescription would not be an effective policy. Fourth, our study was among adults with private health insurance, not covering the population with federal insurance programs, such as Medicaid. This will limit the generalizability of the identified patterns. Lastly, we identified significant associations between opioids and stimulants in this study, while causality and direction of effects cannot be ascertained from the current study design. These limitations highlight areas for future research to provide a more comprehensive understanding of the factors influencing opioid use.

In this large-scale cohort study, we found that stimulant prescriptions had a significant impact on opioid dose trajectories, suggesting a strong association between these two types of drugs across the country. Our results highlight a strong longitudinal association between stimulants and opioids, indicating that stimulant prescriptions were positively associated with increasing opioid prescription dose trajectories. Additionally, our analysis revealed notable gender and regional variations among the four regions of the United States. Our findings suggested that co-prescriptions could serve as a persistent driving force for the twin epidemic in the U.S. healthcare systems over a 10-year period. This evidence needs to be considered in the development of future prescription guidelines and hospital policy.

## Contributors

PZ and WS conceptualized and supervised this study. SL and WS designed the longitudinal cohort study, and PZ accessed, processed, and verified the dataset and methods underlying this study. SL developed the methodology and conducted the data analysis. SL, WS, and PZ wrote the original draft, with critical contributions from DWB and RDU. DWB and RDU provided important clinical guidance. All authors assisted with literature search, data analysis, and data interpretation. All authors from The Ohio State University had full access to all the data in the study. All authors read and approved the final manuscript and had final responsibility for the decision to submit for publication.

## Data sharing statement

The dataset we used is from MarketScan Commercial Claims and Encounters (CCAE, more than 130 million patients from 2012 to 2021), which contains individual-level and de-identified healthcare claims information. Access to the MarketScan data analyzed in this study is provided by The Ohio State University. The dataset is available at https://www.merative.com/real-world-evidence.

## Declaration of interests

DWB reports grants and personal fees from EarlySense, personal fees from CDI Negev, equity from ValeraHealth, equity from Clew, equity from MDClone, personal fees and equity from AESOP, personal fees and equity from FeelBetter, and personal fees and equity from Guided Clinical Solutions, outside the submitted work. RDU serves as a secretary of the Society for Ambulatory Anesthesia (unpaid), a treasurer of the Association of Anesthesia Clinical Directors (unpaid), and a vice president of ERAS USA (unpaid), and reports fees from AcelRx. The other authors declare no competing interests.
