# Supplementary material for: The recent trend of twin epidemic in the United States: a 10-year longitudinal cohort study of co-prescriptions of opioids and stimulants
Source: Lancet Reg Health Am. 2025 Feb 17;44:101030. doi: 10.1016/j.lana.2025.101030 (PMC11876894; doi:10.1016/j.lana.2025.101030)
Supplement: Supplementary Figures and Tables [file mmc1.docx]

**The Recent Trend of Twin Epidemic in the United States: a 10-year Longitudinal Cohort Study of Co-prescriptions of Opioids and Stimulants**

Contents

[Contents 1](#_Toc188477965)

[Data source 2](#_Toc188477966)

[Opioid and stimulant drugs 2](#_Toc188477967)

[Cohort characteristics 3](#_Toc188477968)

[Inclusion and exclusion criteria of study population 4](#_Toc188477969)

[Study cohort 5](#_Toc188477970)

[Statistics 5](#_Toc188477971)

[Supplementary Methods 8](#_Toc188477972)

[Study design 8](#_Toc188477973)

[Study variables 8](#_Toc188477974)

[Opioid trajectory modeling 8](#_Toc188477975)

[Statistical analysis of covariates 9](#_Toc188477976)

[Statistical Analysis 10](#_Toc188477977)

[Opioid baseline model 10](#_Toc188477978)

[Opioid-stimulant model 11](#_Toc188477979)

[Comparison of patient subgroups 16](#_Toc188477980)

[Model variations 18](#_Toc188477981)

[STROBE Statement 19](#_Toc188477982)

[Reference 22](#_Toc188477983)

Data source

Opioid and stimulant drugs

**Supplementary Table 1:** Opioid and stimulant drugs.

|  | Generic Name |
| --- | --- |
| Opioid | Codeine, Dihydrocodeine, Fentanyl, Hydrocodone, Hydromorphone, Levorphanol, Meperidine, Methadone, Morphine, Nalbuphine, Opium, Oxycodone, Oxymorphone, Pentazocine, Propoxyphene, Remifentanil, Sufentanil |
| Stimulant | Amphetamine, Armodafinil, Benzphetamine, Dexfenfluramine, Dexmethylphenidate, Dextroamphetamine, Diethylpropion, Fenfluramine, Levmetamfetamine, Lisdexamfetamine, Mazindol, Methamphetamine, Methylphenidate, Modafinil, Phendimetrazine, Phenmetrazine, Phentermine |

Cohort characteristics

**Supplementary Table 2:** International classification of diseases codes (ICD-9/10) to identify comorbidity.^1–3^

| **Diagnoses** | **ICD-9** | **ICD-10** |
| --- | --- | --- |
| Cancer | 140, 141, 142, 143, 144, 145, 146, 147, 148, 149, 150, 151, 152, 153, 154, 155, 156, 157, 158, 159, 160, 161, 162, 163, 164, 165, 166, 167, 168, 169, 170, 171, 172, 174, 175, 176, 177, 178, 179, 180, 181, 182, 183, 184, 185, 186, 187, 188, 189, 190, 191, 192, 193, 194, 195, 196, 197, 198, 199, 200, 201, 202, 203, 204, 205, 206, 207, 208, 2386 | C00, C01, C02, C03, C04, C05, C06, C07, C08, C09, C10, C11, C12, C13, C14, C15, C16, C17, C18, C19, C20, C21, C22, C23, C24, C25, C26, C30, C31, C32, C33, C34, C37, C38, C39, C40, C41, C43, C45, C46, C47, C48, C49, C50, C51, C52, C53, C54, C55, C56, C57, C58, C60, C61, C62, C63, C64, C65, C66, C67, C68, C69, C70, C71, C72, C73, C74, C75, C76, C77, C78, C79, C80, C81, C82, C83, C84, C85, C88, C90, C91, C92, C93, C94, C95, C96, C97 |
| ADHD | 31400, 31401, 3142 | F90 |
| Depression | 2962, 2963, 3004, 311 | F32, F33 |
| Anxiety | 3000, 3001, 3002, 3003, 3083, 30921, 30981 | F40, F41, F42, F43, F44, F45, F48 |
| Bipolar | 2960, 2961, 2964, 2965, 2966, 2967, 2968, 2969 | F30, F31, F34, F39 |
| Schizophrenia/Psychoses | 29510, 2952, 29530, 29560, 29570, 29590, 2970, 2971, 2972, 2973, 2983, 2984, 2988, 2989, 30122 | F20, F21, F22, F23, F24, F25, F28, F29 |
| Opioid overdose | 96500, 96501, 96502, 96509, E8500, E8501, E8502, E9500 | T400, T401, T402, T403, T404, T405X2, T405X2A, T405X2D, T405X2S, T40602, T40602A, T40602D, T40602S, T40692A, T40692S, T407X2A, T407X2D, T408X2A, T40902A, T40992A, T40992D |
| Opioid use disorder | 3040, 3047, 3055 | F11 |
| Alcohol use disorder | 291, 303, 3050 | F10 |
| Cannabis use disorder | 3043, 3052 | F12 |
| Tobacco use disorder | 3051 | F17, Z720 |
| Other substance use disorder | 292, 3041, 3042, 3044, 3045, 3046, 3048, 3049, 3053, 3054, 3056, 3057, 3058, 3059 | F13, F14, F15, F16, F18, F19 |

Inclusion and exclusion criteria of study population

**Supplementary Figure 1:** **Patient inclusion and exclusion criteria.**


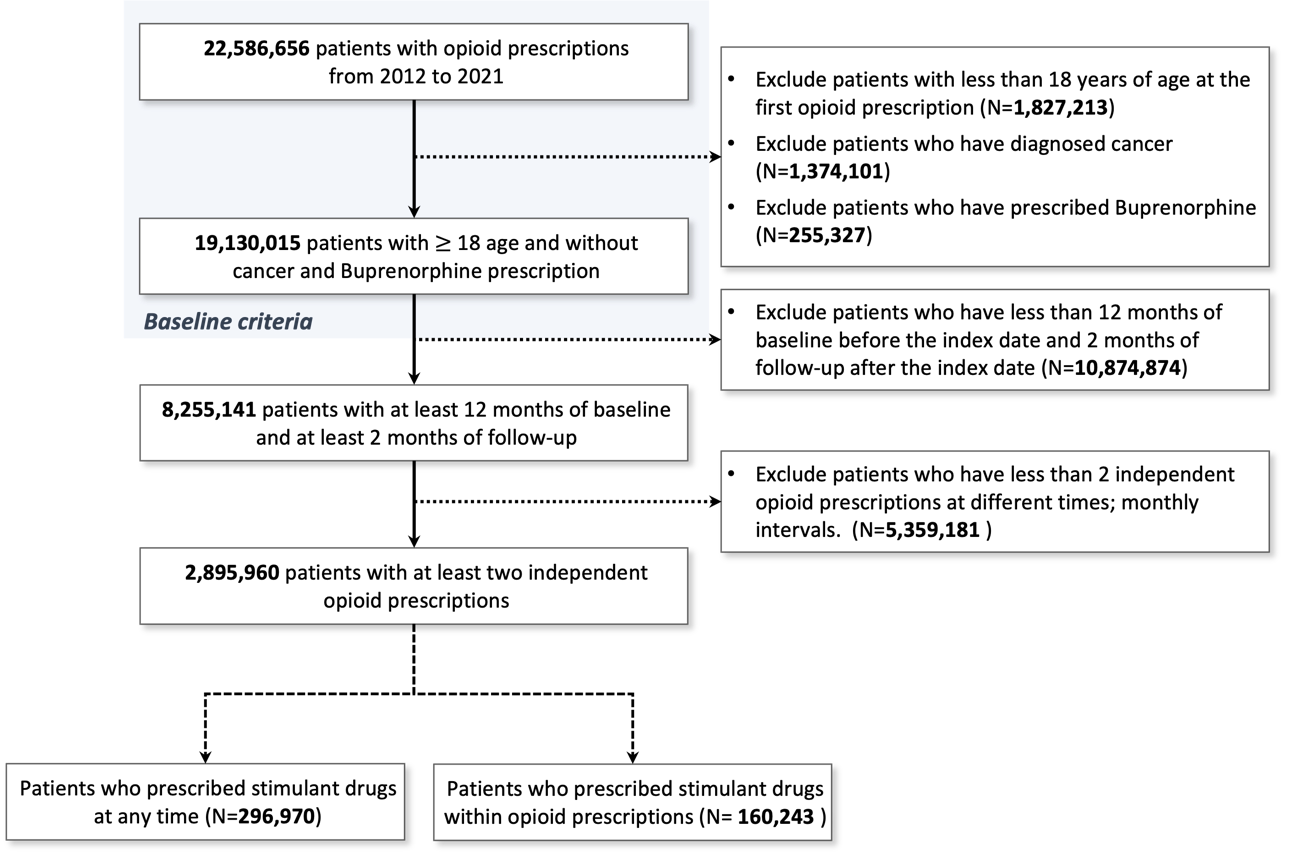


Study cohort

Statistics

**Supplementary Figure 2: A) Age distribution at the index date, B) Distribution of Avg MME, and C) Distribution of Total MME**, presented for: (a) The study cohort; (b) The distribution by gender; (c) The distribution by patient groups with and without stimulant prescriptions. The index date=the first date of opioid prescriptions, Avg MME=the average of the mean daily MMEs for months in which opioids were dispensed, Total MME=the sum of all the mean daily MMEs.

**
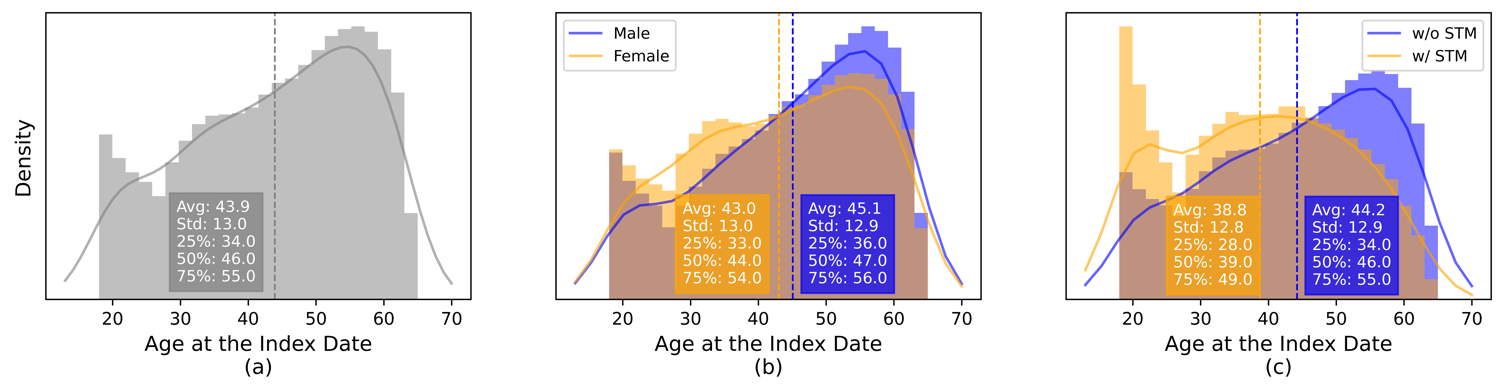
**

**A)**


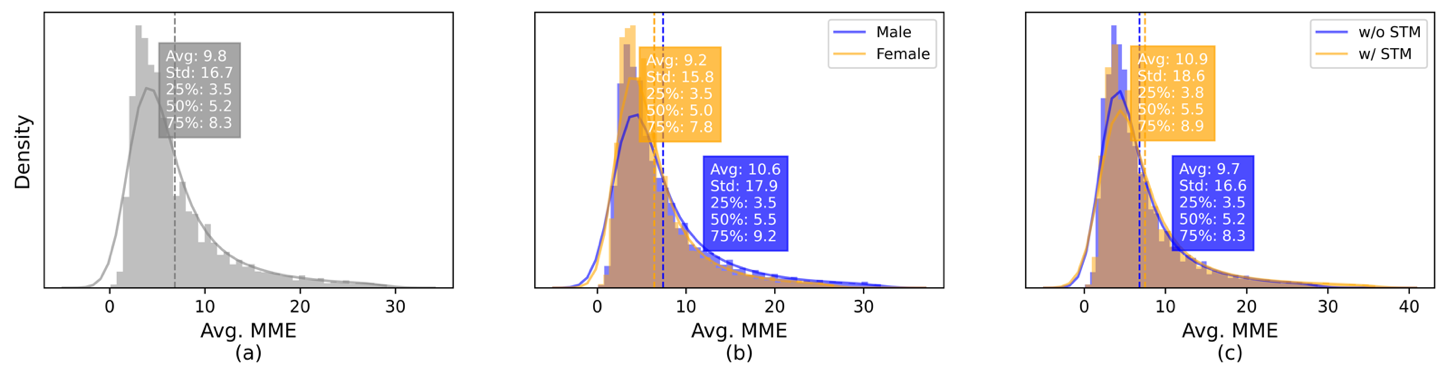


**B)**


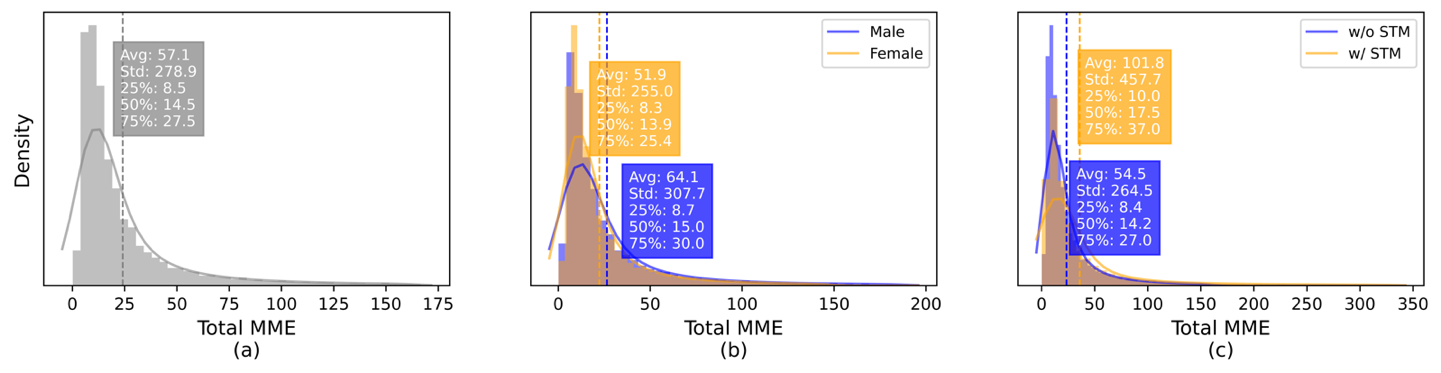


**C)**

**Supplementary Figure 3: Distribution of patients' study duration before and after the index date.** The index date=the first date of opioid prescriptions.


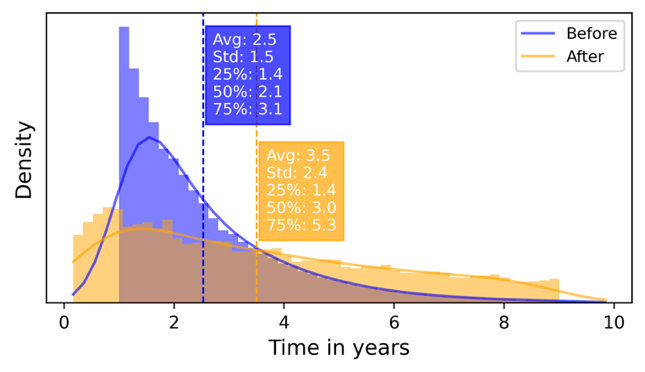


**Supplementary Table 3:** Summary statistics of the study cohort by U.S. region.

|  | |  | **Region** | | | |
| --- | --- | --- | --- | --- | --- | --- |
| **Measure** | | **Total** | **Northeast** | **North Central** | **South** | **West** |
| Patient Demo | Patient No (%) | 2895960 | 370408 (12.8) | 584685 (20.2) | 1440593 (49.7) | 469963 (16.2) |
|  | Female No (%) | 1651883 (57.0) | 200003 (54.0) | 324517 (55.5) | 847402 (58.8) | 262174 (55.8) |
|  | Age, median | 46 (34-55) | 47 (35-56) | 46 (34-55) | 45 (34-55) | 45 (33-55) |
| Opioid Prsc | Prsc. No, mean | 4.0 (5.8) | 3.7 (5.1) | 4.0 (5.5) | 4.2 (6.4) | 3.8 (5.0) |
|  | Prsc. No, median | 3 (2-4) | 2 (2-4) | 3 (2-4) | 3 (2-4) | 3 (2-4) |
|  | Avg MME, mean | 9.8 (16.7) | 9.2 (16.0) | 9.5 (15.4) | 9.0 (15.0) | 12.9 (22.3) |
|  | Avg MME, median | 5.4 (3.6-9.2) | 5.3 (3.5-8.8) | 5.4 (3.5-9.3) | 5.4 (3.6-8.9) | 5.8 (3.8-10.8) |
|  | Total MME, mean | 57.1 (278.9) | 50.5 (255.3) | 50.2 (216.1) | 59.3 (312.0) | 62.7 (257.0) |
|  | Total MME, median | 15.0 (8.8-31.7) | 14.2 (8.3-28.0) | 15.0 (8.5-32.2) | 15.0 (9.0-30.5) | 16.3 (9.2-37.5) |
| Stimulant Prsc | Patient^i^ No (%) | 160243 (5.5) | 17006 (4.6) | 31680 (5.4) | 89993 (6.2) | 20215 (4.3) |
|  | Prsc No^ii^, mean | 12.4 (16.3) | 12.7 (17.0) | 12.7 (16.5) | 12.6 (16.4) | 11.4 (15.2) |
|  | Prsc No, median | 6 (2-16) | 6 (2-16) | 6 (2-16) | 6 (2-16) | 6 (2-14) |
| Comorbidities^iii^ | Depression No. (%) | 281463 (9.7) | 36717 (9.9) | 63473 (10.9) | 129777 (9.0) | 47484 (10.1) |
|  | Anxiety | 352879 (12.2) | 48335 (13.0) | 72403 (12.4) | 173901 (12.1) | 54144 (11.5) |
|  | Bipolar | 56612 (2.0) | 8284 (2.2) | 12346 (2.1) | 25806 (1.8) | 9495 (2.0) |
|  | Schizophrenia/Psychoses | 7668 (0.3) | 1209 (0.3) | 1646 (0.3) | 3473 (0.2) | 1226 (0.3) |
|  | Chronic Acute Pain | 1669571 (57.7) | 222618 (60.1) | 346045 (59.2) | 812796 (56.4) | 267434 (56.9) |
|  | ADHD | 76217 (2.6) | 7887 (2.1) | 14923 (2.5) | 42836 (3.0) | 9682 (2.1) |
|  | Opioid overdose | 785 (0.03) | 99 (0.0) | 192 (0.0) | 334 (0.0) | 142 (0.0) |
|  | Opioid use disorder | 6397 (0.2) | 1063 (0.3) | 978 (0.2) | 3269 (0.2) | 961 (0.2) |
|  | Tobacco use disorder | 180710 (6.2) | 22575 (6.1) | 45972 (7.9) | 88681 (6.2) | 20767 (4.4) |
|  | Alcohol use disorder | 33638 (1.2) | 5594 (1.5) | 7880 (1.4) | 13451 (0.9) | 6344 (1.4) |
|  | Cannabis use disorder | 9939 (0.3) | 1518 (0.4) | 2748 (0.5) | 3790 (0.3) | 1810 (0.4) |
|  | Other substance use disorder | 12983 (0.4) | 2031 (0.5) | 2338 (0.4) | 6300 (0.4) | 2113 (0.4) |
| Comorbidities^iv^ | Depression | 591356 (20.4) | 72684 (19.6) | 130081 (22.2) | 290205 (20.1) | 93154 (19.8) |
|  | Anxiety | 881093 (30.4) | 117600 (31.7) | 181026 (31.0) | 445309 (30.9) | 131572 (28.0) |
|  | Bipolar | 142969 (4.9) | 21112 (5.7) | 31145 (5.3) | 67851 (4.7) | 21875 (4.7) |
|  | Schizophrenia/Psychoses | 18992 (0.7) | 2854 (0.8) | 4086 (0.7) | 8783 (0.6) | 3088 (0.7) |
|  | Chronic Acute Pain | 2325814 (80.3) | 304849 (82.3) | 476720 (81.5) | 1152298 (80.0) | 369144 (78.5) |
|  | ADHD | 136857 (4.7) | 14867 (4.0) | 26438 (4.5) | 76764 (5.3) | 17640 (3.8) |
|  | Opioid overdose | 5280 (0.2) | 641 (0.2) | 1204 (0.2) | 2573 (0.2) | 831 (0.2) |
|  | Opioid use disorder | 31597 (1.1) | 4621 (1.2) | 5092 (0.9) | 17333 (1.2) | 4301 (0.9) |
|  | Tobacco use disorder | 372575 (12.9) | 47951 (12.9) | 92296 (15.8) | 187157 (13.0) | 41374 (8.8) |
|  | Alcohol use disorder | 90460 (3.1) | 14689 (4.0) | 20840 (3.6) | 38883 (2.7) | 15480 (3.3) |
|  | Cannabis use disorder | 33543 (1.2) | 5007 (1.4) | 9198 (1.6) | 13343 (0.9) | 5841 (1.2) |
|  | Other substance use disorder | 42070 (1.5) | 6541 (1.8) | 7841 (1.3) | 21079 (1.5) | 6248 (1.3) |

#### Data are n (%), median (IQR), or mean. Demo=demographics, No=number, Prsc=prescription, IQR=interquartile range, STM= stimulant, Avg MME=the average of all the mean daily MMEs for months in which opioids were dispensed, Total MME=the sum of all the mean daily MMEs, ADHD=Attention-deficit/hyperactivity disorder.

^i^Patients with and without stimulant prescriptions were identified based on whether they were prescribed stimulants at any time between the start and end dates of their opioid prescriptions.

^i^Stimulant Prsc No was measures between the start and end dates of opioid prescriptions for each individual patient.

^iii^Comorbidities were assessed within one year prior to the index date.

^iv^Comorbidities were assessed after the index date.

Supplementary Methods

Study design

In this retrospective cohort study, we used large-scale longitudinal patient-level healthcare data from the MarketScan Commercial Claims and Encounters (CCAE),^[[1]](#footnote-1)^ which provides some of the largest convenience samples available in proprietary U.S. databases—with over 293 million unique patients since 1995.^[[2]](#footnote-2)^ The database contains de-identified, individual-level healthcare claims on a national scale and is primarily used to evaluate health utilization and services.

Study variables

All opioid prescriptions were converted to oral morphine milligram equivalents (MMEs). We determined the patient's daily dose of opioids in MME (mg/day) by multiplying the strength and quantity of opioids dispensed on the day by published conversion factors,^[[3]](#footnote-3)^ expressed as: strength per unit × (number of units / days supplied) × MME conversion factor. The total opioid MME dose for a defined time interval was estimated as the product of the days treated times the daily dose. For patients prescribed multiple opioids during the time interval, the MMEs of all prescribed opioids were summed. We calculated the monthly MME of all opioids dispensed for every non-overlapped 30-day period from the index date at the individual patient level. The mean daily MME per monthly interval was obtained by dividing the monthly MME by 30 days. The MME values were then scaled to a range of 0-1 by censoring values at a maximum of 300 MME (greater than the 99th percentile) and dividing by 300.

We modeled the MMEs over time at monthly intervals. The primary covariate in our analysis was time, defined as the relative time span over which opioid prescriptions were monitored. Specifically, time 0 was designated as the index month, the first 30-day interval from the index date, with subsequent intervals sequentially numbered. This temporal framework allows for a longitudinal examination of prescription patterns over time. Additionally, we incorporated stimulant prescriptions as a time-varying covariate to control for potential confounding factors that could influence the trajectory of opioid doses. At each time step, we calculated the cumulative number of stimulant prescriptions, spanning from one year before the index date up to that time step, and treated it as a time-varying covariate.

Opioid trajectory modeling

We employed trajectory analysis, a model-based clustering method used in previous studies,^4^ to identify latent patterns of opioid prescription dosage changes over time. The analysis captures time-series information that cannot be modeled using a single cross-sectional measure.^1^ Grouped-based trajectory modeling (GBTM) was used to classify the cohort into multiple distinct longitudinal trajectories (e.g., dosage increase, decrease, or stable over time) based on their opioid dose patterns. The method enables the identification of heterogenous subgroups within a population, where the number and composition of these subpopulations are derived from the data rather than predefined a priori.^5^ All patients were classified into subgroups representing distinct trajectories.

We analyzed the trajectories of monthly opioid doses, defined as the mean daily MME, over a 9-year period after cohort entry using the GBTM model. These data were treated as following a beta distribution. A variety of model configurations were explored, testing the number of groups ranging from 1 to 6 and the degree of polynomials from 1 to 5. The best model was selected using the Bayesian information criterion (BIC), the Bayes factor approximation, and clinically relevant group allocations. BIC reflects the model fit, identifying the most parsimonious model that minimizes unexplained variation.^5^

Statistical analysis of covariates

Patient groups by region, gender, stimulant prescriptions, and trajectory groups were compared statistically using Kruskal-Wallis and Mann-Whitney U tests, with significance defined as *P* < 0·05. The characteristics compared included age, the number of opioid prescriptions, the Avg MME, and the Total MME.

We employed a multivariate logistic regression model estimating trajectory membership to compute odds ratios between different trajectory groups,^1^ evaluating factors associated with each group based on patient demographics, comorbidities within one year prior to the index date, and stimulant prescription characteristics. The stimulant characteristics included: 1) the number of stimulant prescriptions within one year prior to the index date, and 2) the cumulative number of stimulant prescriptions at each monthly time step from one year before the index date up to that time step.

Statistical analyses were performed using the SciPy package in Python (version 3.9.7).

Statistical Analysis

Opioid baseline model

|  | | **Trajectory groups** | | | | |  |
| --- | --- | --- | --- | --- | --- | --- | --- |
| **Measure** | | **Very low dose** | **Low-dose**  **decreasing** | **Low-dose**  **increasing** | **Moderate-dose increasing** | **High-dose**  **sustained use** | ***P* value^v^** |
| Patient Demo | Patient No (%) | 2243167 (77·5) | 204607 (7·0) | 335266 (11·6) | 68504 (2·4) | 44416 (1·5) | ·· |
|  | Female (%) | 1321531 (58·9) | 104429 (51·0) | 168328 (50·2) | 35100 (51·2) | 22495 (50·6) | ·· |
|  | Age, median | 44 (33-54) | 50 (39-57) | 50 (39-58) | 49 (37-57) | 49 (38-57) | < ·0001 |
| Opioid Prsc | Prsc No, mean | 3·205 | 6·664 | 5·774 | 10·325 | 9·662 | < ·0001 |
|  | Prsc No, median | 2 (2-3) | 4 (3-7) | 3 (2-5) | 3 (2-10) | 4 (2-9) | ·· |
|  | Avg MME, mean | 4·907 | 17·950 | 15·957 | 46·861 | 115·427 | < ·0001 |
|  | Avg MME, median | 4·533 (3·25-6·25) | 14·25 (11·16-21·66) | 14·58 (11·90-18·83) | 43·75 (36·11-55·41) | 104·1 (84·16-137·5) | ·· |
|  | Total MME, mean | 16·398 | 108·985 | 104·57 | 467·305 | 884·601 | < ·0001 |
|  | Total MME, median | 12· (7·67-18·5) | 67·5 (41·5-119·1) | 43·33 (28·6-77·4) | 150· (95-400) | 333·3 (225-695·9) | ·· |
| Stimulant Prcs | Patient^i^ No (%) | 119617 (5·3) | 15522 (7·6) | 17106 (5·1) | 4617 (6·7) | 3381 (7·6) | ·· |
|  | Prsc No^ii^, mean | 12·283 | 15·606 | 10·484 | 12·611 | 13·034 | < ·0001 |
|  | Prcs No, median | 6 (2-16) | 8 (3-21) | 5 (2-13) | 6 (2-16) | 7 (2-16) | ·· |
| Comorbidities^iii^ | Depression (%) | 204040 (9·1) | 23708 (11·6) | 37389 (11·2) | 9663 (14·1) | 6663 (15·0) | ·· |
|  | Anxiety | 266208 (11·9) | 25871 (12·6) | 43345 (12·9) | 10604 (15·5) | 6851 (15·4) | ·· |
|  | Bipolar | 42179 (1·9) | 4465 (2·2) | 6961 (2·1) | 1803 (2·6) | 1204 (2·7) | ·· |
|  | Schizophrenia/Psychoses | 5366 (0·2) | 649 (0·3) | 1139 (0·3) | 271 (0·4) | 243 (0·5) | ·· |
|  | Chronic Acute Pain | 1186621 (52·9) | 150561 (73·6) | 251445 (75·0) | 48765 (71·2) | 32179 (72·4) | ·· |
|  | ADHD | 59085 (2·6) | 5524 (2·7) | 8638 (2·6) | 1887 (2·8) | 1083 (2·4) | ·· |
|  | Opioid overdose | 497 (0·02) | 80 (0·04) | 125 (0·04) | 50 (0·07) | 33 (0·07) | ·· |
|  | Opioid use disorder | 3112 (0·1) | 580 (0·3) | 1300 (0·4) | 822 (1·2) | 583 (1·3) | ·· |
|  | Tobacco use disorder | 129021 (5·8) | 15396 (7·5) | 25328 (7·6) | 6574 (9·6) | 4391 (9·9) | ·· |
|  | Alcohol use disorder | 22513 (1·0) | 3331 (1·6) | 5611 (1·7) | 1335 (2·0) | 848 (1·9) | ·· |
|  | Cannabis use disorder | 7552 (0·3) | 693 (0·3) | 1142 (0·3) | 347 (0·5) | 205 (0·5) | ·· |
|  | Other substance use disorder | 8415 (0·4) | 1189 (0·6) | 1962 (0·6) | 792 (1·2) | 625 (1·4) | ·· |
| Comorbidities^iv^ | Depression (%) | 438574 (19·6) | 55667 (27·2) | 68106 (20·3) | 16839 (24·6) | 12170 (27·4) | ·· |
|  | Anxiety | 677249 (30·2) | 75738 (37·0) | 91871 (27·4) | 21431 (31·3) | 14804 (33·3) | ·· |
|  | Bipolar | 107827 (4·8) | 13643 (6·7) | 15111 (4·5) | 3683 (5·4) | 2705 (6·1) | ·· |
|  | Schizophrenia/Psychoses | 13602 (0·6) | 1963 (1·0) | 2285 (0·7) | 617 (0·9) | 525 (1·2) | ·· |
|  | Chronic Acute Pain | 1736760 (77·4) | 191341 (93·5) | 299567 (89·4) | 58630 (85·6) | 39516 (89·0) | ·· |
|  | ADHD | 106129 (4·7) | 11361 (5·6) | 14181 (4·2) | 3122 (4·6) | 2064 (4·7) | ·· |
|  | Opioid overdose | 2974 (0·1) | 830 (0·4) | 854 (0·3) | 340 (0·5) | 282 (0·6) | ·· |
|  | Opioid use disorder | 13385 (0·6) | 4800 (2·4) | 7043 (2·1) | 3784 (5·5) | 2585 (5·8) | ·· |
|  | Tobacco use disorder | 272502 (12·1) | 35977 (17·6) | 44854 (13·4) | 11281 (16·5) | 7961 (17·9) | ·· |
|  | Alcohol use disorder | 64443 (2·9) | 10240 (5·0) | 11395 (3·4) | 2549 (3·7) | 1833 (4·1) | ·· |
|  | Cannabis use disorder | 25667 (1·1) | 3110 (1·5) | 3272 (1·0) | 875 (1·3) | 619 (1·4) | ·· |
|  | Other substance use disorder | 27196 (1·2) | 5105 (2·5) | 5787 (1·7) | 2218 (3·2) | 1764 (4·0) | ·· |

**Supplementary Table 4:** Descriptive characteristics by trajectory groups from the opioid baseline model.

#### Data are n (%), median (IQR), or mean. Demo=demographics, No=number, Prsc=prescription, IQR=interquartile range, STM=stimulant, Avg MME=the average of all the mean daily MMEs for months in which opioids were dispensed, Total MME=the sum of all the mean daily MMEs, ADHD=Attention-deficit/hyperactivity disorder.

^i^Patients with stimulant prescriptions were identified based on whether they were prescribed stimulants at any time between the start and end dates of their opioid prescriptions.

^ii^Stimulant Prcs No was measured between the start and end dates of opioid prescriptions for each individual patient.

^iii^Comorbidities were assessed within one year prior to the index date.

^iv^Comorbidities were assessed after the index date.

^v^Kruskal-Wallis tests were performed to compare all trajectory groups.

Opioid-stimulant model

**Supplementary Table 5:** Descriptive characteristics from the opioid-stimulant model, stratified by region, on demographic characteristics.

|  |  |  | **Trajectory groups** |  |  |  |  |  |
| --- | --- | --- | --- | --- | --- | --- | --- | --- |
| **Measure** | **Region** | **Total** | **Very low dose** | **Low-dose**  **decreasing** | **Low-dose**  **increasing** | **Moderate-dose increasing** | **High-dose**  **sustained use** | ***P* value^i^** |
| Patient No (%) | Total | 2895960 | 2245684 (77·5) | 295527 (10·2) | 245072 (8·5) | 68248 (2·4) | 41429 (1·4) | ·· |
|  | Northeast | 370408 (12·8) | 294634 (13·1) | 34093 (11·5) | 29632 (12·1) | 7673 (11·2) | 4376 (10·6) | ·· |
|  | North Central | 584685 (20·2) | 452316 (20·1) | 60164 (20·4) | 51287 (20·9) | 13612 (19·9) | 7306 (17·6) | ·· |
|  | South | 1440593 (49·7) | 1133880 (50·5) | 153477 (51·9) | 114979 (46·9) | 22500 (33·0) | 15757 (38·0) | ·· |
|  | West | 469963 (16·2) | 343119 (15·3) | 45145 (15·3) | 44761 (18·3) | 23399 (34·3) | 13539 (32·7) | ·· |
|  | Unknown | 30311 (1·0) | 21735 (0·1) | 2648 (0·9) | 4413 (1·8) | 1064 (1·6) | 451 (1·1) | ·· |
| Female (%) | Total | 1651883 (57·0) | 1322171 (58·9) | 151186 (51·2) | 122151 (49·8) | 35311 (51·7) | 21064 (50·8) | ·· |
|  | Northeast | 200003 (54·0) | 164085 (55·7) | 16157 (47·4) | 13869 (46·8) | 3843 (50·1) | 2049 (46·8) | ·· |
|  | North Central | 324517 (55·5) | 258980 (57·3) | 29414 (48·9) | 25123 (49·0) | 7080 (52·0) | 3920 (53·7) | ·· |
|  | South | 847402 (58·8) | 687957 (60·7) | 81346 (53·0) | 58559 (50·9) | 11356 (50·5) | 8184 (51·9) | ·· |
|  | West | 262174 (55·8) | 197820 (57·7) | 22818 (50·5) | 22306 (49·8) | 12529 (53·5) | 6701 (49·5) | ·· |
| Age, median | Total | 46 (34-55) | 44 (33-54) | 50 (39-57) | 50 (39-58) | 48 (37-57) | 49 (38-56) | < ·0001 |
|  | Northeast | 47 (35-56) | 46 (34-55) | 51 (41-57) | 51 (41-58) | 49 (38-57) | 51 (40-57) | < ·0001 |
|  | North Central | 46 (34-55) | 44 (32-55) | 51 (40-57) | 51 (40-59) | 48 (37-56) | 48 (37-56) | < ·0001 |
|  | South | 45 (34-55) | 44 (33-54) | 49 (39-56) | 50 (39-58) | 50 (40-57) | 49 (38-56) | < ·0001 |
|  | West | 45 (33-55) | 44 (32-54) | 49 (38-57) | 49 (36-57) | 46 (34-56) | 49 (38-57) | < ·0001 |

Data are n (%), or median (IQR). No=number, IQR=interquartile range.

^i^Kruskal-Wallis tests were performed to compare all trajectory groups.

**Supplementary Table 6:** Descriptive characteristics from the opioid-stimulant model, stratified by region, on stimulant prescription characteristics.

|  |  |  | **Trajectory groups** |  |  |  |  |  |
| --- | --- | --- | --- | --- | --- | --- | --- | --- |
| **Measure** | **Region** | **Total** | **Very low dose** | **Low-dose**  **decreasing** | **Low-dose**  **increasing** | **Moderate-dose increasing** | **High-dose**  **sustained use** | ***P* value^iii^** |
| STM patient^i^ No (%) | Total | 160243 (5·5) | 111087 (4·9) | 14793 (5·0) | 23616 (9·6) | 6681 (9·8) | 4066 (9·8) | ·· |
|  | Northeast | 17006 (4·6) | 12626 (4·3) | 1355 (4·0) | 2221 (7·5) | 517 (6·7) | 287 (6·6) | ·· |
|  | North Central | 31680 (5·4) | 22517 (5·0) | 2763 (4·6) | 4524 (8·8) | 1167 (8·6) | 709 (9·7) | ·· |
|  | South | 89993 (6·2) | 62934 (5·6) | 8945 (5·8) | 13678 (12·0) | 3069 (14·0) | 1367 (8·7) | ·· |
|  | West | 20215 (4·3) | 12205 (3·6) | 1661 (3·7) | 2881 (6·4) | 1828 (7·8) | 1640 (12·0) | ·· |
| STM Prsc No^ii^, mean | Total | 12·438 | 12·330 | 15·700 | 10·878 | 12·192 | 13·009 | < ·0001 |
|  | Northeast | 12·657 | 12·709 | 16·114 | 10·238 | 11·714 | 14·460 | < ·0001 |
|  | North Central | 12·718 | 12·635 | 16·108 | 11·361 | 12·284 | 11·520 | < ·0001 |
|  | South | 12·603 | 12·347 | 15·838 | 11·066 | 13·886 | 15·725 | < ·0001 |
|  | West | 11·435 | 11·626 | 14·232 | 10·263 | 9·674 | 11·198 | < ·0001 |
| STM Prsc No, median | Total | 6 (2-16) | 6 (2-16) | 8 (3-21) | 5 (2-14) | 6 (2-15) | 7 (2-16) | ·· |
|  | Northeast | 6 (2-16) | 6 (2-16) | 8 (3-21) | 5 (2-12) | 6 (2-13) | 8 (3-19) | ·· |
|  | North Central | 6 (2-16) | 6 (3-16) | 9 (3-22) | 6 (2-14) | 6 (2-15) | 6 (2-13) | ·· |
|  | South | 6 (2-16) | 6 (2-16) | 8 (3-21) | 6 (2-14) | 6 (2-18) | 8 (3-20) | ·· |
|  | West | 6 (2-14) | 6 (2-15) | 7 (3-19) | 5 (2-12) | 5 (2-11) | 6 (2-14) | ·· |

Data are n (%), median (IQR), mean. STM=stimulant, No=number, Prsc=prescription, IQR=interquartile range.

^i^Patients with stimulant prescriptions were identified based on whether they were prescribed stimulants at any time between the start and end dates of their opioid prescriptions.

^ii^Stimulant Prsc No was measured between the start and end dates of opioid prescriptions for each individual patient.

^iii^Kruskal-Wallis tests were performed to compare all trajectory groups.

**Supplementary Table 7:** Descriptive characteristics from the opioid-stimulant model, stratified by region, on opioid prescription characteristics.

|  |  |  | **Trajectory groups** |  |  |  |  |  |
| --- | --- | --- | --- | --- | --- | --- | --- | --- |
| **Measure** | **Region** | **Total** | **Very low dose** | **Low-dose decreasing** | **Low-dose  increasing** | **Moderate-dose increasing** | **High-dose**  **sustained use** | ***P* Value^i^** |
| Prsc No, mean | Total | 4·014 | 3·148 | 6·343 | 6·399 | 10·304 | 9·916 | < ·0001 |
|  | Northeast | 3·696 | 2·991 | 5·89 | 5·648 | 9·47 | 10·715 | < ·0001 |
|  | North Central | 3·946 | 3·172 | 6·549 | 6·096 | 7·902 | 7·945 | < ·0001 |
|  | South | 4·188 | 3·191 | 6·527 | 7·242 | 16·966 | 12·591 | < ·0001 |
|  | West | 3·804 | 3·107 | 5·861 | 5·079 | 5·5 | 7·446 | < ·0001 |
| Prsc No, median | Total | 3 (2-4) | 2 (2-3) | 4 (3-7) | 3 (2-5) | 3 (2-10) | 4 (2-9) | ·· |
|  | Northeast | 2 (2-4) | 2 (2-3) | 4 (2-6) | 3 (2-5) | 3 (2-10) | 5 (3-12) | ·· |
|  | North Central | 3 (2-4) | 2 (2-3) | 4 (3-7) | 3 (2-5) | 3 (2-6) | 3 (2-7) | ·· |
|  | South | 3 (2-4) | 2 (2-3) | 4 (3-7) | 3 (2-6) | 6 (2-24) | 4 (2-13) | ·· |
|  | West | 3 (2-4) | 2 (2-3) | 4 (2-6) | 3 (2-4) | 3 (2-4) | 3 (2-7) | ·· |
| Avg MME, mean | Total | 9·795 | 4·933 | 15·332 | 18·042 | 51·682 | 116·075 | < ·0001 |
|  | Northeast | 9·227 | 4·877 | 15·081 | 17·861 | 52·262 | 122·587 | < ·0001 |
|  | North Central | 9·517 | 4·861 | 15·039 | 18·099 | 52·481 | 112·012 | < ·0001 |
|  | South | 8·989 | 4·959 | 15·338 | 17·635 | 48·721 | 117·282 | < ·0001 |
|  | West | 12·935 | 4·962 | 15·794 | 19·147 | 53·98 | 113·982 | < ·0001 |
| Avg MME, median | Total | 5·416 (3·58-9·17) | 4·55 (3·25-6·25) | 12·77 (10·66-17·5) | 16·5 (13·33-21·52) | 48·55 (40-60·83) | 108·3 (83·33-141·6) | ·· |
|  | Northeast | 5·25 (3·49-8·75) | 4·5 (3·15-6·25) | 12·70 (10·70-17·09) | 16·25 (13·33-21·25) | 49·16 (40-62·25) | 116 (91-150) | ·· |
|  | North Central | 5·375 (3·50-9·25) | 4·444 (3·17-6·25) | 12·70 (10·67-17) | 16·66 (13·41-21·56) | 50 (40-61·66) | 106·2 (83·72-134·6) | ·· |
|  | South | 5·416 (3·61-8·88) | 4·583 (3·31-6·29) | 12·75 (10·58-17·5) | 16·20 (13·25-20·87) | 45 (38-56·66) | 105 (75·90-150) | ·· |
|  | West | 5·833 (3·75-10·83) | 4·555 (3·33-6·33) | 13·07 (10·83-18·16) | 17·5 (13·75-23·33) | 50 (41·66-63·75) | 108·3 (87·5-134·1) | ·· |
| Total MME, mean | Total | 57·129 | 15·956 | 91·161 | 129·713 | 489·836 | 904·017 | < ·0001 |
|  | Northeast | 50·49 | 14·982 | 83·75 | 112·903 | 467·665 | 1027·984 | < ·0001 |
|  | North Central | 50·176 | 15·931 | 92·242 | 121·134 | 374·498 | 721·465 | < ·0001 |
|  | South | 59·315 | 16·239 | 93·906 | 148·348 | 787·385 | 1132·773 | < ·0001 |
|  | West | 62·66 | 15·785 | 86·478 | 103·395 | 273·699 | 671·796 | < ·0001 |
| Total MME, median | Total | 15· (8·83-31·66) | 12· (7·67-18·66) | 56·66 (35·26-99·16) | 50 (32·5-90·5) | 168·3 (107·5-435) | 335·8 (228·7-708·3) | ·· |
|  | Northeast | 14·16 (8·33-28) | 11·5 (7·5-17·5) | 52·83 (33·75-91) | 47·33 (31·57-82·5) | 171·6 (105-434·5) | 416·1 (250-1050) | ·· |
|  | North Central | 15 (8·53-32·16) | 11·66 (7·5-18·5) | 57·5 (35·83-100) | 50 (33·33-89) | 156·6 (100-300) | 316·6 (225-577·0) | ·· |
|  | South | 15 (9-30·5) | 12·3 (8-19) | 57·5 (35·83-101·7) | 50 (32·5-98·33) | 275 (120-1035) | 325 (215·5-881·6) | ·· |
|  | West | 16·25 (9·17-37·53) | 11·66 (7·67-18·33) | 55 (35-95) | 50 (33·75-83) | 148·3 (100-233·3) | 337·5 (237·5-580) | ·· |

Data are mean or median (IQR). Prsc=prescription, No=number; IQR=interquartile range, Avg MME=the average of all the mean daily MMEs for months in which opioids were dispensed, Total MME=the sum of all the mean daily MMEs.

^i^Kruskal-Wallis tests were performed to compare all trajectory groups.

|  |  |  | **Trajectory groups** | | | | |
| --- | --- | --- | --- | --- | --- | --- | --- |
| **Measure** | **Region** | **Total** | **Very low dose** | **Low-dose**  **decreasing** | **Low-dose**  **increasing** | **Moderate-dose increasing** | **High-dose**  **sustained use** |
| Depression | Total | 281463 (9·72) | 202919 (9·04) | 32545 (11·0) | 29636 (12·1) | 10040 (14·7) | 6323 (15·3) |
|  | Northeast | 36717 (9·91) | 27527 (9·34) | 3839 (11·3) | 3451 (11·6) | 1145 (14·9) | 755 (17·3) |
|  | North Central | 63473 (10·9) | 46505 (10·3) | 7002 (11·6) | 6627 (12·9) | 2094 (15·4) | 1245 (17·0) |
|  | South | 129777 (9·01) | 94558 (8·34) | 16186 (10·5) | 13424 (11·7) | 3464 (15·4) | 2145 (13·6) |
|  | West | 47484 (10·1) | 31762 (9·26) | 5152 (11·4) | 5399 (12·1) | 3107 (13·3) | 2064 (15·2) |
| Anxiety | Total | 352879 (12·2) | 265442 (11·8) | 36079 (12·2) | 33912 (13·8) | 10993 (16·1) | 6453 (15·6) |
|  | Northeast | 48335 (13·0) | 37661 (12·8) | 4445 (13·0) | 4088 (13·8) | 1316 (17·2) | 825 (18·9) |
|  | North Central | 72403 (12·4) | 55186 (12·2) | 7123 (11·8) | 6816 (13·3) | 2154 (15·8) | 1124 (15·4) |
|  | South | 173901 (12·1) | 131834 (11·6) | 18930 (12·3) | 16658 (14·5) | 4077 (18·1) | 2402 (15·2) |
|  | West | 54144 (11·5) | 38060 (11·1) | 5212 (11·5) | 5640 (12·6) | 3230 (13·8) | 2002 (14·8) |
| Bipolar | Total | 56612 (1·95) | 41772 (1·86) | 6010 (2·03) | 5744 (2·34) | 1924 (2·82) | 1162 (2·8) |
|  | Northeast | 8284 (2·24) | 6427 (2·18) | 773 (2·27) | 718 (2·42) | 239 (3·11) | 127 (2·90) |
|  | North Central | 12346 (2·11) | 9295 (2·05) | 1263 (2·10) | 1181 (2·30) | 379 (2·78) | 228 (3·12) |
|  | South | 25806 (1·79) | 19168 (1·69) | 2914 (1·90) | 2693 (2·34) | 660 (2·93) | 371 (2·35) |
|  | West | 9495 (2·02) | 6448 (1·88) | 986 (2·18) | 1036 (2·31) | 614 (2·62) | 411 (3·04) |
| Schizophrenia/  Psychoses | Total | 7668 (0·27) | 5363 (0·24) | 885 (0·30) | 910 (0·37) | 287 (0·42) | 223 (0·54) |
|  | Northeast | 1209 (0·33) | 879 (0·30) | 120 (0·35) | 132 (0·45) | 42 (0·55) | 36 (0·82) |
|  | North Central | 1646 (0·28) | 1157 (0·26) | 182 (0·30) | 209 (0·41) | 60 (0·44) | 38 (0·52) |
|  | South | 3473 (0·24) | 2439 (0·22) | 433 (0·28) | 399 (0·35) | 112 (0·50) | 90 (0·57) |
|  | West | 1226 (0·26) | 815 (0·24) | 135 (0·30) | 154 (0·34) | 70 (0·299) | 52 (0·38) |
| Chronic acute pain | Total | 1669571 (57·7) | 1187764 (52·9) | 216649 (73·3) | 186744 (76·2) | 48310 (70·8) | 30104 (72·7) |
|  | Northeast | 222618 (60·1) | 164791 (55·9) | 25920 (76·0) | 22904 (77·3) | 5613 (73·2) | 3390 (77·5) |
|  | North Central | 346045 (59·2) | 245333 (54·2) | 45583 (75·8) | 40048 (78·1) | 9751 (71·6) | 5330 (73·0) |
|  | South | 812796 (56·4) | 585019 (51·6) | 110471 (72·0) | 88444 (76·9) | 17651 (78·4) | 11211 (71·1) |
|  | West | 267434 (56·9) | 179219 (52·2) | 32511 (72·0) | 31628 (70·7) | 14323 (61·2) | 9753 (72·0) |
| ADHD | Total | 76217 (2·63) | 56259 (2·51) | 5908 (2·0) | 10181 (4·15) | 2611 (3·83) | 1258 (3·04) |
|  | Northeast | 7887 (2·13) | 6111 (2·07) | 583 (1·71) | 891 (3·01) | 216 (2·82) | 86 (1·97) |
|  | North Central | 14923 (2·55) | 11214 (2·48) | 1043 (1·73) | 1847 (3·6) | 540 (3·97) | 279 (3·82) |
|  | South | 42836 (2·97) | 31776 (2·8) | 3483 (2·27) | 5936 (5·16) | 1174 (5·22) | 467 (2·96) |
|  | West | 9682 (2·06) | 6564 (1·91) | 740 (1·64) | 1321 (2·95) | 650 (2·78) | 407 (3·01) |
| Opioid overdose | Total | 785 (0·03) | 501 (0·02) | 100 (0·03) | 99 (0·04) | 52 (0·08) | 33 (0·08) |
|  | Northeast | 99 (0·03) | 74 (0·03) | 10 (0·03) | 8 (0·03) | 3 (0·04) | 4 (0·09) |
|  | North Central | 192 (0·03) | 128 (0·03) | 20 (0·03) | 27 (0·05) | 14 (0·10) | 3 (0·04) |
|  | South | 334 (0·02) | 202 (0·02) | 45 (0·03) | 47 (0·04) | 22 (0·10) | 18 (0·11) |
|  | West | 142 (0·03) | 91 (0·03) | 23 (0·05) | 12 (0·03) | 12 (0·051) | 4 (0·03) |
| Opioid use disorder | Total | 6397 (0·22) | 3080 (0·14) | 758 (0·26) | 1194 (0·49) | 833 (1·22) | 532 (1·28) |
|  | Northeast | 1063 (0·29) | 597 (0·20) | 125 (0·37) | 155 (0·52) | 107 (1·39) | 79 (1·81) |
|  | North Central | 978 (0·17) | 567 (0·13) | 94 (0·16) | 166 (0·32) | 91 (0·67) | 60 (0·82) |
|  | South | 3269 (0·23) | 1455 (0·13) | 412 (0·27) | 675 (0·59) | 472 (2·1) | 255 (1·62) |
|  | West | 961 (0·20) | 435 (0·13) | 114 (0·25) | 170 (0·38) | 133 (0·57) | 109 (0·81) |
| Tobacco use disorder | Total | 180710 (6·24) | 129032 (5·75) | 21780 (7·37) | 19272 (7·86) | 6555 (9·6) | 4071 (9·83) |
|  | Northeast | 22575 (6·09) | 16167 (5·49) | 2490 (7·3) | 2370 (8·0) | 922 (12·0) | 626 (14·3) |
|  | North Central | 45972 (7·86) | 33277 (7·36) | 5459 (9·07) | 4841 (9·44) | 1503 (11·0) | 892 (12·2) |
|  | South | 88681 (6·16) | 64417 (5·68) | 11197 (7·3) | 9224 (8·02) | 2407 (10·7) | 1436 (9·11) |
|  | West | 20767 (4·42) | 13369 (3·90) | 2355 (5·22) | 2406 (5·38) | 1588 (6·79) | 1049 (7·75) |
| Alcohol use disorder | Total | 33638 (1·16) | 22534 (1·00) | 4583 (1·55) | 4409 (1·8) | 1325 (1·94) | 787 (1·9) |
|  | Northeast | 5594 (1·51) | 3880 (1·32) | 666 (1·95) | 714 (2·41) | 180 (2·35) | 154 (3·52) |
|  | North Central | 7880 (1·35) | 5323 (1·18) | 1098 (1·83) | 998 (1·95) | 280 (2·06) | 181 (2·48) |
|  | South | 13451 (0·93) | 9113 (0·80) | 1940 (1·26) | 1781 (1·55) | 417 (1·85) | 200 (1·27) |
|  | West | 6344 (1·35) | 3992 (1·16) | 838 (1·86) | 848 (1·89) | 420 (1·79) | 246 (1·82) |
| Cannabis use disorder | Total | 9939 (0·34) | 7533 (0·34) | 949 (0·32) | 920 (0·38) | 338 (0·50) | 199 (0·48) |
|  | Northeast | 1518 (0·41) | 1174 (0·40) | 125 (0·37) | 123 (0·42) | 56 (0·73) | 40 (0·91) |
|  | North Central | 2748 (0·47) | 2090 (0·46) | 255 (0·42) | 258 (0·50) | 89 (0·65) | 56 (0·77) |
|  | South | 3790 (0·26) | 2930 (0·26) | 378 (0·25) | 347 (0·30) | 83 (0·37) | 52 (0·33) |
|  | West | 1810 (0·39) | 1291 (0·38) | 182 (0·40) | 179 (0·4) | 108 (0·46) | 50 (0·37) |
| Other substance use disorder | Total | 12983 (0·45) | 8350 (0·37) | 1569 (0·53) | 1636 (0·67) | 831 (1·22) | 597 (1·44) |
|  | Northeast | 2031 (0·55) | 1379 (0·47) | 230 (0·68) | 226 (0·76) | 117 (1·52) | 79 (1·81) |
|  | North Central | 2338 (0·40) | 1623 (0·36) | 262 (0·44) | 269 (0·52) | 99 (0·73) | 85 (1·16) |
|  | South | 6300 (0·44) | 3901 (0·34) | 814 (0·53) | 847 (0·74) | 428 (1·9) | 310 (1·97) |
|  | West | 2113 (0·45) | 1354 (0·40) | 235 (0·52) | 272 (0·61) | 157 (0·67) | 95 (0·70) |

**Supplementary Table 8:** Descriptive characteristics from the opioid-stimulant model, stratified by region, on comorbidities within one year prior to the index date.

Data are n (%). ADHD=Attention-deficit/hyperactivity disorder.

**Supplementary Table 9:** Descriptive characteristics from the opioid-stimulant model, stratified by region, on comorbidities after the index date.

|  |  |  | **Trajectory groups** |  |  |  |  |
| --- | --- | --- | --- | --- | --- | --- | --- |
| **Measure** | **Region** | **Total** | **Very low dose** | **Low-dose**  **decreasing** | **Low-dose**  **increasing** | **Moderate-dose increasing** | **High-dose**  **sustained use** |
| Depression | Total | 591356 (20·4) | 433467 (19·3) | 75912 (25·7) | 52576 (21·5) | 17744 (26·0) | 11657 (28·1) |
|  | Northeast | 72684 (19·6) | 55430 (18·8) | 8341 (24·5) | 5938 (20·0) | 1803 (23·5) | 1172 (26·8) |
|  | North Central | 130081 (22·2) | 96922 (21·4) | 16178 (26·9) | 11474 (22·4) | 3477 (25·5) | 2030 (27·8) |
|  | South | 290205 (20·1) | 214296 (18·9) | 39975 (26·0) | 25291 (22·0) | 6377 (28·3) | 4266 (27·1) |
|  | West | 93154 (19·8) | 63510 (18·5) | 10885 (24·1) | 8942 (20·0) | 5778 (24·7) | 4039 (29·8) |
| Anxiety | Total | 881093 (30·4) | 670977 (29·9) | 104314 (35·3) | 69254 (28·3) | 22316 (32·7) | 14232 (34·4) |
|  | Northeast | 117600 (31·7) | 93389 (31·7) | 12397 (36·4) | 8209 (27·7) | 2218 (28·9) | 1387 (31·7) |
|  | North Central | 181026 (31·0) | 139153 (30·8) | 21053 (35·0) | 14216 (27·7) | 4313 (31·7) | 2291 (31·4) |
|  | South | 445309 (30·9) | 340939 (30·1) | 55649 (36·3) | 34701 (30·2) | 8312 (36·9) | 5708 (36·2) |
|  | West | 131572 (28·0) | 93802 (27·3) | 14642 (32·4) | 11235 (25·1) | 7187 (30·7) | 4706 (34·8) |
| Bipolar | Total | 142969 (4·94) | 106133 (4·73) | 18075 (6·12) | 12133 (4·95) | 3961 (5·8) | 2667 (6·44) |
|  | Northeast | 21112 (5·70) | 16497 (5·60) | 2329 (6·83) | 1564 (5·28) | 459 (5·98) | 263 (6·01) |
|  | North Central | 31145 (5·33) | 23363 (5·17) | 3864 (6·42) | 2664 (5·19) | 788 (5·79) | 466 (6·38) |
|  | South | 67851 (4·71) | 50581 (4·46) | 9105 (5·93) | 5727 (4·98) | 1451 (6·45) | 987 (6·26) |
|  | West | 21875 (4·65) | 15064 (4·39) | 2664 (5·9) | 2014 (4·5) | 1216 (5·2) | 917 (6·77) |
| Schizophrenia/  Psychoses | Total | 18992 (0·66) | 13425 (0·60) | 2576 (0·87) | 1861 (0·76) | 629 (0·92) | 501 (1·21) |
|  | Northeast | 2854 (0·77) | 2114 (0·72) | 355 (1·04) | 254 (0·86) | 74 (0·964) | 57 (1·3) |
|  | North Central | 4086 (0·70) | 2911 (0·64) | 548 (0·91) | 400 (0·78) | 139 (1·02) | 88 (1·2) |
|  | South | 8783 (0·61) | 6208 (0·55) | 1219 (0·79) | 884 (0·77) | 249 (1·11) | 223 (1·42) |
|  | West | 3088 (0·66) | 2067 (0·60) | 436 (0·97) | 305 (0·68) | 157 (0·671) | 123 (0·91) |
| Chronic acute pain | Total | 2325814 (80·3) | 1734406 (77·2) | 276009 (93·4) | 219610 (89·6) | 58738 (86·1) | 37051 (89·4) |
|  | Northeast | 304849 (82·3) | 235404 (79·9) | 32200 (94·4) | 26783 (90·4) | 6539 (85·2) | 3923 (89·6) |
|  | North Central | 476720 (81·5) | 355041 (78·5) | 56932 (94·6) | 46560 (90·8) | 11805 (86·7) | 6382 (87·4) |
|  | South | 1152298 (80·0) | 870788 (76·8) | 142941 (93·1) | 104219 (90·6) | 20322 (90·3) | 14028 (89·0) |
|  | West | 369144 (78·5) | 258085 (75·2) | 41563 (92·1) | 38132 (85·2) | 19073 (81·5) | 12291 (90·8) |
| ADHD | Total | 136857 (4·73) | 101355 (4·51) | 12337 (4·17) | 16583 (6·77) | 4250 (6·23) | 2332 (5·63) |
|  | Northeast | 14867 (4·01) | 11644 (3·95) | 1182 (3·47) | 1532 (5·17) | 333 (4·34) | 176 (4·02) |
|  | North Central | 26438 (4·52) | 19826 (4·38) | 2249 (3·74) | 3111 (6·07) | 818 (6·01) | 434 (5·94) |
|  | South | 76764 (5·33) | 57148 (5·04) | 7289 (4·75) | 9526 (8·28) | 1921 (8·54) | 880 (5·58) |
|  | West | 17640 (3·75) | 11951 (3·48) | 1545 (3·42) | 2198 (4·91) | 1127 (4·82) | 819 (6·05) |
| Opioid overdose | Total | 5280 (0·18) | 2937 (0·13) | 1024 (0·346) | 674 (0·28) | 361 (0·53) | 284 (0·69) |
|  | Northeast | 641 (0·17) | 379 (0·13) | 121 (0·355) | 82 (0·28) | 38 (0·495) | 21 (0·48) |
|  | North Central | 1204 (0·21) | 714 (0·16) | 250 (0·416) | 139 (0·271) | 60 (0·441) | 41 (0·561) |
|  | South | 2573 (0·18) | 1425 (0·13) | 481 (0·313) | 334 (0·29) | 192 (0·853) | 141 (0·895) |
|  | West | 831 (0·18) | 410 (0·12) | 166 (0·368) | 112 (0·25) | 66 (0·282) | 77 (0·569) |
| Opioid  use disorder | Total | 31597 (1·09) | 12956 (0·58) | 6113 (2·07) | 6189 (2·53) | 3869 (5·67) | 2470 (5·96) |
|  | Northeast | 4621 (1·25) | 2291 (0·78) | 836 (2·45) | 760 (2·56) | 424 (5·53) | 310 (7·08) |
|  | North Central | 5092 (0·87) | 2353 (0·52) | 1031 (1·71) | 928 (1·81) | 487 (3·58) | 293 (4·01) |
|  | South | 17333 (1·2) | 6622 (0·58) | 3396 (2·21) | 3696 (3·21) | 2348 (10·4) | 1271 (8·07) |
|  | West | 4301 (0·92) | 1619 (0·47) | 819 (1·81) | 757 (1·69) | 560 (2·39) | 546 (4·03) |
| Tobacco  use disorder | Total | 372575 (12·9) | 270142 (12·0) | 49778 (16·8) | 33582 (13·7) | 11541 (16·9) | 7532 (18·2) |
|  | Northeast | 47951 (12·9) | 35706 (12·1) | 5814 (17·1) | 4071 (13·7) | 1387 (18·1) | 973 (22·2) |
|  | North Central | 92296 (15·8) | 68095 (15·1) | 12111 (20·1) | 8204 (16·0) | 2474 (18·2) | 1412 (19·3) |
|  | South | 187157 (13·0) | 136730 (12·1) | 26373 (17·2) | 16530 (14·4) | 4540 (20·2) | 2984 (18·9) |
|  | West | 41374 (8·8) | 27114 (7·9) | 5085 (11·3) | 4160 (9·29) | 2945 (12·6) | 2070 (15·3) |
| Alcohol  use disorder | Total | 90460 (3·12) | 63613 (2·83) | 13792 (4·67) | 8619 (3·52) | 2664 (3·9) | 1772 (4·28) |
|  | Northeast | 14689 (3·97) | 10695 (3·63) | 2073 (6·08) | 1335 (4·51) | 331 (4·31) | 255 (5·83) |
|  | North Central | 20840 (3·56) | 14852 (3·28) | 3185 (5·29) | 1921 (3·75) | 560 (4·11) | 322 (4·41) |
|  | South | 38883 (2·7) | 27519 (2·43) | 6197 (4·04) | 3723 (3·24) | 885 (3·93) | 559 (3·55) |
|  | West | 15480 (3·29) | 10212 (2·98) | 2253 (4·99) | 1543 (3·45) | 856 (3·66) | 616 (4·55) |
| Cannabis  use disorder | Total | 33543 (1·16) | 25375 (1·13) | 4068 (1·38) | 2566 (1·05) | 906 (1·33) | 628 (1·52) |
|  | Northeast | 5007 (1·35) | 3891 (1·32) | 565 (1·66) | 369 (1·25) | 104 (1·36) | 78 (1·78) |
|  | North Central | 9198 (1·57) | 7003 (1·55) | 1111 (1·85) | 720 (1·40) | 226 (1·66) | 138 (1·89) |
|  | South | 13343 (0·93) | 10237 (0·90) | 1650 (1·08) | 944 (0·82) | 294 (1·31) | 218 (1·38) |
|  | West | 5841 (1·24) | 4123 (1·2) | 730 (1·62) | 516 (1·15) | 278 (1·19) | 194 (1·43) |
| Other substance use disorder | Total | 42070 (1·45) | 26706 (1·19) | 6493 (2·2) | 4791 (1·95) | 2355 (3·45) | 1725 (4·16) |
|  | Northeast | 6541 (1·77) | 4302 (1·46) | 1003 (2·94) | 688 (2·32) | 308 (4·01) | 240 (5·48) |
|  | North Central | 7841 (1·34) | 5318 (1·18) | 1104 (1·83) | 874 (1·70) | 318 (2·34) | 227 (3·11) |
|  | South | 21079 (1·46) | 13056 (1·15) | 3416 (2·23) | 2487 (2·16) | 1245 (5·53) | 875 (5·55) |
|  | West | 6248 (1·33) | 3875 (1·13) | 916 (2·03) | 672 (1·50) | 442 (1·89) | 343 (2·53) |

Data are n (%). ADHD=Attention-deficit/hyperactivity disorder.

Comparison of patient subgroups

**Supplementary Table 10**: Comparison of patient subgroups by gender.

|  | **Measure** | **Total** | **Male** | **Female** | ***P* value^v^** |
| --- | --- | --- | --- | --- | --- |
| **Demographics** | Patient No (%) | 2895960 | 1244077 (43·0) | 1651883 (57·0) | ·· |
|  | Age, mean | 43.92 (13.00) | 45·08 (12·89) | 43·04 (13·02) | < ·0001 |
| **Opioid Prsc** | Prsc No, mean | 4·01 (5·82) | 4·08 (6·08) | 3·96 (5·63) | < ·0001 |
|  | Avg MME, mean | 9·80 (16·73) | 10·60 (17·91) | 9·19 (15·75) | < ·0001 |
|  | Total MME, mean | 57·13 (278·89) | 64·11 (307·66) | 51·87 (254·96) | < ·0001 |
| **Stimulant Prsc** | Patient^i^ No (%) | 160243 (5·5) | 56737 (4.6) | 103506 (6.3) | ·· |
|  | Prsc No^ii^, mean | 12·44 (16·28) | 12.91 (16.62) | 12.18 (16.08) | < ·0001 |
| **Comorbidities^iii^** | Depression (%) | 281463 (9·7) | 75534 (6.1) | 205929 (12.5) | ·· |
|  | Anxiety | 352879 (12·2) | 106359 (8.6) | 246520 (14.9) | ·· |
|  | Bipolar | 56612 (1·9) | 17788 (1.4) | 38824 (2.4) | ·· |
|  | Schizophrenia/Psychoses | 7668 (0·3) | 3474 (0.3) | 4194 (0.3) | ·· |
|  | Chronic Acute Pain | 1669571 (57·7) | 707585 (56.9) | 961986 (58.2) | ·· |
|  | ADHD | 76217 (2·6) | 31779 (2.6) | 44438 (2.7) | ·· |
|  | Opioid overdose | 785 (0·03) | 334 (0.03) | 451 (0.03) | ·· |
|  | Opioid use disorder | 6397 (0·2) | 3224 (0.3) | 3173 (0.2) | ·· |
|  | Tobacco use disorder | 180710 (6·2) | 90526 (7.3) | 90184 (5.5) | ·· |
|  | Alcohol use disorder | 33638 (1·2) | 21072 (1.7) | 12566 (0.8) | ·· |
|  | Cannabis use disorder | 9939 (0·3) | 5969 (0.5) | 3970 (0.2) | ·· |
|  | Other substance use disorder | 12983 (0·4) | 6780 (0.5) | 6203 (0.4) | ·· |
| **Comorbidities^iv^** | Depression (%) | 591356 (20·4) | 168248 (13.5) | 423108 (25.6) | ·· |
|  | Anxiety | 881093 (30·4) | 277903 (22.3) | 603190 (36.5) | ·· |
|  | Bipolar | 142969 (4·9) | 45610 (3.7) | 97359 (5.9) | ·· |
|  | Schizophrenia/Psychoses | 18992 (0·7) | 8389 (0.7) | 10603 (0.6) | ·· |
|  | Chronic Acute Pain | 2325814 (80·3) | 991176 (79.7) | 1334638 (80.8) | ·· |
|  | ADHD | 136857 (4·7) | 54732 (4.4) | 82125 (5.0) | ·· |
|  | Opioid overdose | 5280 (0·2) | 2214 (0.2) | 3066 (0.2) | ·· |
|  | Opioid use disorder | 31597 (1·1) | 15717 (1.3) | 15880 (1.0) | ·· |
|  | Tobacco use disorder | 372575 (12·9) | 189768 (15.3) | 182807 (11.1) | ·· |
|  | Alcohol use disorder | 90460 (3·1) | 54955 (4.4) | 35505 (2.2) | ·· |
|  | Cannabis use disorder | 33543 (1·2) | 19095 (1.5) | 14448 (0.9) | ·· |
|  | Other substance use disorder | 42070 (1·5) | 21565 (1.7) | 20505 (1.2) | ·· |

#### Data are n (%) or mean (SD). No=number, Prsc=prescription, IQR=interquartile range, Avg MME=the average of the mean daily MMEs for months in which opioids were dispensed, Total MME=the sum of all the mean daily MMEs.

#### ^i^Patients with stimulant prescriptions were identified based on whether they were prescribed stimulants at any time between the start and end dates of their opioid prescriptions.

^ii^Stimulant Prsc No was measured between the start and end dates of opioid prescriptions for each individual patient.

^iii^Comorbidities were assessed within one year prior to the index date.

^iv^Comorbidities were assessed after the index date.

^v^Mann-Whitney U tests were performed to compare gender groups.

**Supplementary Table 11**: Comparison of patient subgroups by region.

|  |  | Northeast | |  | North Central | |  | South | |  | West | |  |  |
| --- | --- | --- | --- | --- | --- | --- | --- | --- | --- | --- | --- | --- | --- | --- |
|  | **Total** |  | *P* value^iii^ |  |  | *P* value^iii^ |  |  | *P* value^iii^ |  |  | *P* value^iii^ |  | *P* value^iv^ |
| **Demographics** | |  |  |  |  |  |  |  |  |  |  |  |  |  |
| Patient No (%) | 2895960 | 370408 (12·8) | ·· |  | 584685 (20·2) | ·· |  | 1440593 (49·7) | ·· |  | 469963 (16·2) |  |  |  |
| Age | 43.92 (13·00) | 44.63 (13·04) | < ·0001 |  | 44.01 (13·28) | < ·0001 |  | 43.79 (12·84) | < ·0001 |  | 43.55 (13·09) | < ·0001 |  | < ·0001 |
| **Opioid Prsc** | |  |  |  |  |  |  |  |  |  |  |  |  |  |
| Prsc No | 4.01 (5.82) | 3.70 (5·06) | < ·0001 |  | 3.95 (5·47) | 0·06 |  | 4.19 (6·38) | < ·0001 |  | 3.80 (5·03) | < ·0001 |  | < ·0001 |
| Avg MME | 9.80 (16.72) | 9.23 (15·97) | < ·0001 |  | 9.52 (15·42) | < ·0001 |  | 8.99 (15·03) | < ·0001 |  | 12.93 (22·31) | < ·0001 |  | < ·0001 |
| Total MME | 57.1 (278.9) | 50.49 (255·25) | < ·0001 |  | 50.18 (216·05) | < ·0001 |  | 59.31 (312·02) | ·0006 |  | 62.66 (256·99) | < ·0001 |  | < ·0001 |
| **Stimulant Prsc** | |  |  |  |  |  |  |  |  |  |  |  |  |  |
| Patient^i^ No (%) | 160243 (5.5) | 17006 (4.6) | ·· |  | 31680 (5.4) | ·· |  | 89993 (6.2) | ·· |  | 20215 (4.3) | ·· |  | ·· |
| Prsc No^ii^ | 12·44 (16·30) | 12·66 (17·00) | 0·99 |  | 12·72 (16·49) | <·0001 |  | 12·60 (16·38) | ·0002 |  | 11·43 (15·19) | < ·0001 |  | < ·0001 |

Data are n (%) or mean (SD). No=number, Prsc=prescription, Avg MME=the average of the mean daily MMEs for months in which opioids were dispensed, Total MME=the sum of all the mean daily MMEs.

#### ^i^Patients with stimulant prescriptions were identified based on whether they were prescribed stimulants at any time between the start and end dates of their opioid prescriptions.

^ii^Stimulant Prsc No was measured between the start and end dates of opioid prescriptions for each individual patient.

^iii^Mann-Whitney U tests were performed to compare each region with all other regions.

^iv^Kruskal-Wallis tests were performed to compare differences across all regions.

Model variations

**Supplementary Table 12**: Model variations with different numbers of trajectory groups and polynomial orders.

|  | Number of Trajectory Groups | | | | | |
| --- | --- | --- | --- | --- | --- | --- |
| Degree | 1 | 2 | 3 | 4 | 5 | 6 |
| 1 | -54128659 | -58307857 | -62895614 | -63839756 | -65984391 | -65214961 |
| 2 | -54140458 | -58350196 | -63008980 | -63839648 | -65267829 | -65879090 |
| 3 | -54147210 | -58316913 | -62913297 | -63716135 | **-66053602** | -65227815 |
| 4 | -54153566 | -58302933 | -62840651 | -64112761 | -65940042 | -65532496 |
| 5 | -54153181 | -58317960 | -62872006 | -63754410 | -65454933 | -65360912 |

Data represent Bayesian Information Criterion (BIC), with lower values indicating better model fit. BIC values were calculated based on the log-likelihood of the model and penalized by the number of model parameters associated with the trajectory groups and polynomial orders.

STROBE Statement

**Supplementary Table 13**: STROBE Statement—Checklist of items that should be included in reports of cohort studies

|  | Item No | Recommendation |  |
| --- | --- | --- | --- |
| **Title and abstract** | 1 | (*a*) Indicate the study’s design with a commonly used term in the title or the abstract | Y  (Title) |
|  |  | (*b*) Provide in the abstract an informative and balanced summary of what was done and what was found | Y  (Abstract) |
| Introduction | | |  |
| Background/rationale | 2 | Explain the scientific background and rationale for the investigation being reported | Y  (Introduction) |
| Objectives | 3 | State specific objectives, including any prespecified hypotheses | Y  (Introduction) |
| Methods | | |  |
| Study design | 4 | Present key elements of study design early in the paper | Y  (Study design,  Participants) |
| Setting | 5 | Describe the setting, locations, and relevant dates, including periods of recruitment, exposure, follow-up, and data collection | Y  (Study Design,  Participants) |
| Participants | 6 | (*a*) Give the eligibility criteria, and the sources and methods of selection of participants. Describe methods of follow-up | Y  (Participants) |
|  |  | (*b*) For matched studies, give matching criteria and number of exposed and unexposed | NA |
| Variables | 7 | Clearly define all outcomes, exposures, predictors, potential confounders, and effect modifiers. Give diagnostic criteria, if applicable | Y  (Study variables) |
| Data sources/ measurement | 8* | For each variable of interest, give sources of data and details of methods of assessment (measurement). Describe comparability of assessment methods if there is more than one group | Y  (Study variables,  Cohort characteristics,  Statistical analysis of covariates) |
| Bias | 9 | Describe any efforts to address potential sources of bias | Y  (Study Design,  Participants) |
| Study size | 10 | Explain how the study size was arrived at | Y  (Participants) |
| Quantitative variables | 11 | Explain how quantitative variables were handled in the analyses. If applicable, describe which groupings were chosen and why | Y  (Study variables,  Opioid trajectory modeling) |
| Statistical methods | 12 | (*a*) Describe all statistical methods, including those used to control for confounding | Y  (Statistical analysis of covariates,  Opioid trajectory modeling) |
|  |  | (*b*) Describe any methods used to examine subgroups and interactions | Y  (Opioid trajectory modeling) |
|  |  | (*c*) Explain how missing data were addressed | Y  (Study design) |
|  |  | (*d*) If applicable, explain how loss to follow-up was addressed | Y  (Participants) |
|  |  | (*e*) Describe any sensitivity analyses | Y  (Opioid trajectory modeling) |
| Results | | |  |
| Participants | 13* | (a) Report numbers of individuals at each stage of study—eg numbers potentially eligible, examined for eligibility, confirmed eligible, included in the study, completing follow-up, and analysed | Y  (Descriptive summary of study cohort,  Supplementary Figure 1) |
|  |  | (b) Give reasons for non-participation at each stage | Y  (Supplementary Figure 1) |
|  |  | (c) Consider use of a flow diagram | Y  (Supplementary Figure 1) |
| Descriptive data | 14* | (a) Give characteristics of study participants (eg demographic, clinical, social) and information on exposures and potential confounders | Y  (Descriptive summary of study cohort, Table 1) |
|  |  | (b) Indicate number of participants with missing data for each variable of interest | NA |
|  |  | (c) Summarise follow-up time (eg, average and total amount) | Y  (Descriptive summary of study cohort) |
| Outcome data | 15* | Report numbers of outcome events or summary measures over time | Y  (Descriptive summary of study cohort, Table 1) |
| Main results | 16 | (*a*) Give unadjusted estimates and, if applicable, confounder-adjusted estimates and their precision (eg, 95% confidence interval). Make clear which confounders were adjusted for and why they were included | Y  (Figure 2) |
|  |  | (*b*) Report category boundaries when continuous variables were categorized | NA |
|  |  | (*c*) If relevant, consider translating estimates of relative risk into absolute risk for a meaningful time period | NA |
| Other analyses | 17 | Report other analyses done—eg analyses of subgroups and interactions, and sensitivity analyses | Y  (Supplementary Table 12) |
| Discussion | | |  |
| Key results | 18 | Summarise key results with reference to study objectives | Y  (Discussion) |
| Limitations | 19 | Discuss limitations of the study, taking into account sources of potential bias or imprecision. Discuss both direction and magnitude of any potential bias | Y  (Discussion) |
| Interpretation | 20 | Give a cautious overall interpretation of results considering objectives, limitations, multiplicity of analyses, results from similar studies, and other relevant evidence | Y  (Discussion) |
| Generalisability | 21 | Discuss the generalisability (external validity) of the study results | Y  (Discussion) |
| Other information | | |  |
| Funding | 22 | Give the source of funding and the role of the funders for the present study and, if applicable, for the original study on which the present article is based | Y  (Role of the funding source,  Acknowledgments) |

*Give information separately for exposed and unexposed groups.

Reference

1 Wilson JD, Abebe KZ, Kraemer K, *et al.* Trajectories of opioid use following first opioid prescription in opioid-naive youths and young adults. *JAMA Netw Open* 2021; **4**: e214552–e214552.

2 Zhang P, Tossone K, Ashmead R, *et al.* Examining differences in retention on medication for opioid use disorder: An analysis of Ohio Medicaid data. *J Subst Abuse Treat* 2022; **136**: 108686.

3 Binswanger IA, Shetterly SM, Xu S, *et al.* Opioid dose trajectories and associations with mortality, opioid use disorder, continued opioid therapy, and health plan disenrollment. *JAMA Netw Open* 2022; **5**: e2234671–e2234671.

4 Nguena Nguefack HL, Pagé MG, Katz J, *et al.* Trajectory Modelling Techniques Useful to Epidemiological Research: A Comparative Narrative Review of Approaches. *Clin Epidemiol* 2020; **Volume 12**: 1205–22.

5 Gisev N, Buizen L, Hopkins RE, *et al.* Five-year trajectories of prescription opioid use. *JAMA Netw Open* 2023; **6**: e2328159–e2328159.

1. <https://www.merative.com/real-world-evidence> [↑](#footnote-ref-1)
2. <https://www.merative.com/content/dam/merative/documents/brief/marketscan-research-databases-for-life-sciences-researchers.pdf> [↑](#footnote-ref-2)
3. <https://medicaid.utah.gov/Documents/files/Opioid-Morphine-EQ-Conversion-Factors.pdf> [↑](#footnote-ref-3)
